# Sensory-Related Industrial Additives in the US Packaged Food Supply

**DOI:** 10.3389/fnut.2021.762814

**Published:** 2022-01-13

**Authors:** Marilyn Tseng, Camille J. Grigsby, Abigail Austin, Samir Amin, Aydin Nazmi

**Affiliations:** ^1^Department of Kinesiology and Public Health, California Polytechnic State University, San Luis Obispo, CA, United States; ^2^Department of Food Science and Nutrition, California Polytechnic State University, San Luis Obispo, CA, United States

**Keywords:** artificial food colors, emulsifiers, industrial additives, ingredient list, sweeteners, ultra-processed food, USDA branded food products database

## Abstract

**Background:** Increasing evidence suggests that ultra-processed foods (UPFs) lead to elevated risk of obesity-related conditions, but UPF measurement has been criticized for its subjectivity and lack of clarity on biological mechanism. Sensory-related industrial additives (SRIAs) are a defining feature of UPFs and may encourage overconsumption by enhancing the sensory quality of foods. However, practical challenges have prevented systematic incorporation of SRIAs into UPF measurement.

**Objective:** The objectives of this work were to describe a new, open-source ingredient list search method and to apply this method to describe the presence of SRIAs in US packaged foods.

**Methods:** We developed computer coding to search for 64 common SRIAs related to sweetness, flavor, appearance, and texture in 241,688 foods in the US Branded Food Products Database (BFPD). The BFPD includes manufacturer-provided ingredient lists for ~300,000 branded and private label food items. We determined the total number of SRIAs (0–64) and the number of different types of SRIAs (sweetness, flavor, appearance, texture, 0–4) in each food, then calculated the percent of all foods with SRIAs. This was done for all foods, and by food group for 224,098 items with food group data.

**Results:** Most (64.9%) foods in the BFPD contained at least one SRIA, and more than a third had at least three. Sweets (89.5%), beverages (84.9%), and ready-to-eat (RTE) foods (82.0%) were the most likely to contain SRIAs. With respect to SRIA types, 25.7% of all food items had at least three of the four types of SRIAs examined, with texture-related additives being the most common. Among sweets, 20% had all four types of SRIAs.

**Discussion:** This work confirms the high prevalence of SRIAs in US packaged foods. They are ubiquitous in sweets, beverages, and RTE foods, but also present in substantial proportions of other food groups. Quantifying the presence of SRIAs in ingredient lists offers a novel way to identify UPFs for research; to distinguish more vs. less ultra-processed foods; and to test whether UPFs increase risk for obesity-related conditions through additives that enhance the product's sensory qualities.

## Introduction

A steadily growing body of evidence links highly or “ultra-processed foods” (UPFs) to cardiometabolic conditions and cancer (1–6). Existing frameworks for identifying foods as “ultra-processed” variously consider extent of modification from a food's original form; its ingredients (e.g., the number and types of additives); the use of industrial processing methods, as opposed to methods used in home or culinary preparations; and the purpose of processing (e.g., food safety, convenience, palatability) (7, 8).

However, processed food classification systems have been challenged for their subjectivity, inconsistency, ambiguity, and lack of clarity on biological mechanisms specific to UPFs (7, 9). Such criticisms raise doubts about the validity of findings linking UPF consumption to disease risk, and about implications for policy decisions relating to UPFs. Current systems also treat UPFs as a binary characteristic, such that a flavored yogurt might fall into the same category as a food made entirely of extracted substances. A less subjective way to classify foods that also allows for distinguishing levels of ultra-processing would be a useful complement to current classification frameworks, allow for more quantitative explorations of associations between UPFs and disease, and potentially serve as a useful basis to guide consumers and policy-makers.

The presence of industrial additives as ingredients figures prominently in most major frameworks to classify UPFs (7, 8, 10) and offers a potentially more objective indicator of one component of ultra-processing (11). A food additive is defined broadly as any substance added to food to perform a specific function (12, 13)—for example, to improve safety, slow spoilage, improve or maintain nutritional value, as well as “to improve taste, texture and appearance” (12). Despite their recognized importance to the safety, shelf-life, and nutritional value of foods (12, 14), additives have also been implicated in a variety of health outcomes (15–17). However, additives that enhance the sensory qualities of foods are of particular relevance if UPFs are thought to increase risk by encouraging overconsumption and contributing to excess adiposity (18, 19). Thus, we focus on *sensory-related* industrial additives (SRIAs) as “classes of additives whose function is to […] give the final product sensory properties especially attractive to see, taste, smell and/or touch” (11). Quantifying SRIAs in foods provides a means to evaluate one mechanism by which UPFs have been suggested to increase risk for obesity-related conditions.

SRIAs are a defining feature of UPFs but have not been systematically incorporated into measurement of UPFs in research because of practical challenges in accessing and searching ingredient lists for variably expressed or easily misspelled ingredients. The relatively recent release of a publicly accessible database with ingredient lists for packaged foods presents the opportunity to explore a new method of identifying and describing the presence of SRIAs in packaged foods in the US. The objectives of this paper are: (1) to describe a new method that searches ingredient lists for common SRIAs affecting four aspects of food: sweetness, flavor (other than sweetness), texture, and appearance; and (2) to apply this method to describe the presence of SRIAs in US packaged foods.

## Materials and Methods

### Identification of SRIAs

**Figure 1:**
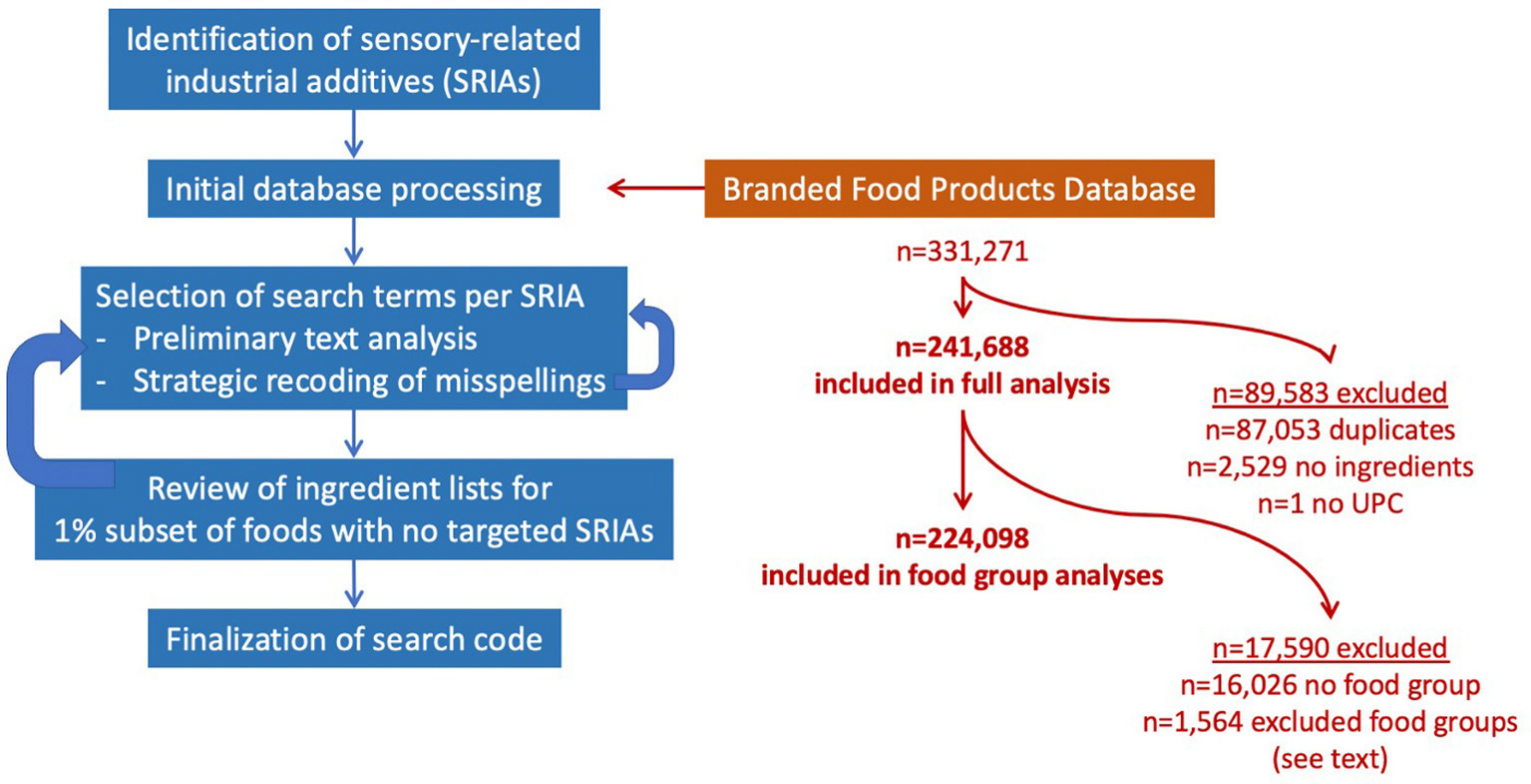
Graphical scheme illustrating study approach and database used. Initial identification of sensory-related industrial additives (SRIAs) was compiled from US Food and Drug Administration (12), International Food Information Council (14), and SugarScience (20) websites. Following preliminary editing of the Branded Food Products Database, an iterative process was used to identify search terms for each SRIA, which included a preliminary text analysis and selective recoding of misspellings. Ingredient lists for a 1% subset of foods with no SRIAs were reviewed for errors or omissions, and search terms were revised. The process was repeated until no further modifications were deemed necessary. Also shown are numbers of BFPD food items included in analyses.

A graphical scheme illustrating our approach is shown in Figure 1. We compiled an initial list of 42 common SRIAs from the US Food and Drug Administration (12) and International Food Information Council (14), including only ingredients and additives specified as sweeteners (nutritive and non-nutritive), color additives, flavors or flavor enhancers, fat replacers, emulsifiers, stabilizers, thickeners, binders, texturizers, and that were also deemed not to be commonly used in domestic or culinary settings (e.g., vanilla extract, pectin). We used the primary function assigned by these websites to categorize each additive as relating to: texture (including emulsifiers, thickeners, and fat replacers), flavor (flavoring agents or flavor enhancers, other than sweetness), sweetness, or appearance (colors, dyes, and glazing agents) (Note that for the remainder of the paper, our use of the term “flavor” excludes sweetness as a flavor). We identified 13 additional different names for sugars in ingredient lists (20) and consulted with an expert in culinary science and food technology (SA) to confirm that the list included sweeteners used primarily in industrial and not in domestic or culinary settings.

We also added to our list seven SRIAs that occurred in at least 0.5% of the 126,556 food products in a recent analysis of French food and beverage products (21). Subsequent iterative verifications of the completeness of our search (described below) resulted in the addition of two more SRIAs to our search. For these last nine SRIAs, we assigned primary function based on online resources (22, 23). Our final list of 64 SRIAs included 21 related to texture, six related to flavor (excluding sweetness), 26 related to sweetness (including 20 nutritive and six non-nutritive), and eleven related to appearance (all colors and dyes except carnauba wax) (Table 1).

**Table 1:** List of sensory-related industrial additives (SRIA) sought in ingredient lists.

**Function**	**Additive**	**E number^**a**^**
**Texture**		
	1. Lecithin	E322
	2. Mono- and diglycerides	E471
	3. Polysorbates	E432-E436
	4. Sorbitan monostearate	E491
	5. Di-, tri-, poly-, and pyro-phosphates	E450-E452
	6. Polyglycerol polyricinoleate	E476
	7. Sodium stearoyl-2-lactylate	E481
	8. Ammonium phosphatides	E442
	9. Sodium caseinate	
	10. Guar gum	E412
	11. Carrageenan	E407
	12. Xanthan gum	E415
	13. Locust (carob) bean gum	E410
	14. Gum arabic (acacia gum)	E414
	15. Hydrogenated oils	
	16. Cellulose, cellulose gum, cellulose gel, crystalline cellulose, carboxymethylcellulose	E460, E466
	17. Modified food starch	E1401
	18. Alginic acid and alginates	E400-E405
	19. Olestra	
	20. Polydextrose	E1200
	21. Whey protein concentrate	
**Flavor (excluding sweetness)**		
	1. Artificial flavor	
	2. Natural flavor	
	3. Disodium guanylate	E627
	4. Disodium inosinate	E631
	5. Autolyzed yeast extract	
	6. Hydrolyzed vegetable proteins	
**Sweetness**		
	1. High fructose corn syrup	
	2. Sorbitol	E420
	3. Mannitol	E421
	4. Xylitol	E967
	5. Erythritol	E968
	6. Maltitol	E965
	7. Polyglycitol syrup	E964
	8. Cane juice	
	9. Corn syrup solids	
	10. Dextrin	E1400
	11. Dextrose	
	12. Fructose	
	13. Fruit juice concentrate	
	14. Glucose	
	15. Invert sugar	
	16. Maltodextrin	
	17. Maltol	E636
	18. Refiner's syrup	
	19. Rice syrup	
	20. Maltose	
	21. Acesulfame potassium	E950
	22. Aspartame	E951
	23. Neotame	E961
	24. Saccharin	E954
	25. Stevia	E960
	26. Sucralose	E955
**Appearance**
	1. FD&C blue no. 1	E133
	2. FD&C blue no. 2	E132
	3. FD&C green no. 3	E143
	4. FD&C red no. 3	E127
	5. FD&C red no. 40	E129
	6. FD&C yellow no. 5	E102
	7. FD&C yellow no. 6	E110
	8. Artificial color	
	9. Titanium dioxide	E171
	10. Caramel color	E150
	11. Carnauba wax	E903

a *Not all additives that we included in our search have E numbers*.

### Branded Food Products Database

We searched for each SRIA within ingredient lists available in the Branded Food Products Database (BFPD). The BFPD is a publicly available database that includes nutrient information and ingredient lists voluntarily provided by food companies for packaged food items (24). In 2019, Baldridge et al. (25) reported that the database “represents >80% of all food and beverage products sold in the US over the past three years.” Manufacturers and retailers submit data to the database using a portal provided by Label Insight, a company specializing in consumer product label data aggregation and analysis; or by directly synchronizing their data through the GS1 Global Data Synchronization Network, if participating in the network. About 95% of food item data in the BFPD are derived from Label Insight.

We used the December 2019 version, which contained 331,271 items. We excluded 37,033 items with duplicate UPC codes, 50,020 items with duplicate brand and food names, 2,529 items with no ingredients, and one item with no UPC, leaving 241,688 foods in our analysis.

Of 241,688 foods, 225,662 had a field representing 227 food categories. We combined food categories into 79 food groups (representing seven broad food categories) developed by the Economic Research Service to correspond with the 2015 Dietary Guidelines for Americans and to capture information on convenience and processing (26). Because the BFPD often did not provide more specific information to categorize foods more specifically, we collapsed the 79 food groups further; for example, all vegetables were combined into one group that included starchy vegetables, tomatoes, dark green vegetables, other red and orange vegetables, beans, lentils and peas or legumes, and other/mixed vegetables. Four food groups (egg and egg substitutes; tofu and meat substitutes; vitamins and meal supplements; alcohol) had fewer than 700 items, suggesting that foods in those groups were being captured in other categories and that the included foods were not representative of all foods in that group. After excluding those four groups, we ended with 224,098 food items in fifteen food groups (Table 2).

**Table 2:** Description and distribution of 15 food groups (representing seven food categories) used in analysis of Branded Food Products Database food items (*N* = 224,098).

**Food group**	**Description**	***N* (%)**
**Grains**	•Breads and buns •Pastas, noodles, rice •Flours, doughs, crusts •Cereals and cereal products •Stuffing	21,801 (9.7)
**Vegetables**	• Prepared/processed/pre-packaged vegetables • Canned or frozen vegetables or beans	12,782 (5.7)
**Fruits and juices**	• Prepared/processed fruit • Canned or frozen fruit • Fruit juices, concentrates, nectars	8,873 (4.0)
**Milk products**		
Cheese	–	10,290 (4.6)
Milk, cream, yogurt	–	6,613 (3.0)
**Meat and protein**		
Meats	• Prepared/processed beef, pork, or poultry • Frozen meat, poultry, patties, burgers, sausages • Canned meat • Sausages, hot dogs, cold cuts	9,904 (4.4)
Fish and seafood	• Prepared/processed fish • Canned or frozen fish or seafood	4,260 (1.9)
Nuts	• Nuts • Nut and seed butters	1,482 (0.7)
Ready-to-eat foods	• Prepared/packaged/ready-made meals, dishes, sandwiches, salads, and pizzas • Frozen dinners, entrees, sides, breakfast foods • Canned soups and stews	18,005 (8.0)
**Other foods**		
Dressings/condiments	• Dressings, mayonnaise, ketchup, mustard, sauces, dips • Oils, butter, spreads • Pickles • Herbs, spices, seasonings	35,253 (15.7)
**Beverages**		
Coffee and tea	–	2,294 (1.0)
Water	–	2,724 (1.2)
All other non-alcoholic	• Soda, soft drinks • Energy, protein, sports drinks	9,696 (4.3)
Sweets	• Cookies, cakes, pastries, pies, puddings • Frozen desserts • Confections, chocolate, candy • Fruit spreads • Dessert sauces and toppings • Sugar	49,039 (21.9)
Salty snacks	• Chips, pretzels, crackers, popcorn, snack bars	31,082 (13.9)

### Search Strategy and Iteration

We edited the unstructured text in the ingredients field of the BFPD—for example, converting all text to lower case, replacing or standardizing symbols, and removing multiple blanks. Preliminary examinations of ingredient lists revealed substantial variability in the spelling and expression of different additives (for examples of this variability, see Supplementary Table 1). We used the Text Explorer platform in JMP, which breaks unstructured text data (such as text in ingredient lists) into terms (“tokens”), to identify misspellings or alternative spellings for each word for the additives of interest.

After identifying the range of misspellings and alternative spellings, we selected the smallest character string that would identify a given additive, in an iterative process in both JMP and SAS to confirm that searching for that string would not identify unwanted items (i.e., false positives). We recoded other misspellings as necessary using the TRANWORD function in SAS. We then searched for the character string using the INDEX function, which searches for specific character strings in a specified field. Examples of this approach are provided in Supplementary Table 1.

To verify the completeness of our search, we reviewed the ingredient lists of a randomly selected subset of 1% (850–900 foods) of foods identified as not having any of our targeted additives, specifically to identify SRIAs missed by our initial searches, as well as SRIAs of potential interest not on our original list. We revised our SAS code to address omissions and repeated the process of reviewing ingredient lists of randomly selected subsets of foods until we noted no further necessary revisions to our search. The final version of SAS code used to produce the results presented here are available in Supplementary File 1.

### Comparison With Independent Database

To evaluate the completeness and accuracy of our search, we compared our search results to data from Open Food Facts (27). Open Food Facts (OFF) is a publicly available international database of >650,000 food products, including almost 350,000 US items. Data on nutrient content and ingredients are submitted by manufacturers and consumers. Included in OFF is a data field indicating the specific additives present in each food, identified by E number, a number assigned to identify food additives approved for use in the European Union. According to a March 2021 email from S. Gigandet ( stephane@openfoodfacts.org), OFF contributors have developed a multilingual ingredients and additives taxonomy that contains different names and synonyms for additives in many languages. Analysts then use a text parser to analyze ingredient lists and search for specific additives and classes of additives that would identify the food as “ultra-processed,” based on published literature describing the NOVA classification system.

We compared results among 125,502 barcode-matched foods for 43 of our 64 selected SRIAs that had E numbers. For each SRIA, we determined the number of foods identified as having that additive by our search vs. by OFF. We then calculated the proportion of OFF-identified foods that our search missed, and the proportion of foods we identified that OFF missed.

In an initial comparison across the 43 SRIAs, the median percent of foods identified by our search as having a given SRIA missed by OFF was 37.9%. In contrast, the median percent of foods that OFF identified as having a given SRIA missed in our search was 1.9%. For example, of 6,285 foods we identified as containing the colorant FD&C yellow #6, OFF missed 2912 (46.3%). In contrast, our search missed 387 (10.3%) of the 3,769 foods OFF identified as containing FD&C yellow #6. OFF missed at least half of all the foods we identified as containing a given additive for 15 of the 43 SRIAs, and at least 5% of foods for all SRIAs except one. Our search missed at least half of OFF-identified foods for two of the 43 SRIAs, and at least 5% for eleven of the SRIAs.

We looked more closely at eleven SRIAs for which we missed >5% of foods captured by OFF. For each of these eleven SRIAs, we examined a subset of up to 50 foods identified by OFF as having the SRIA but not identified by our search, specifically examining their ingredient lists to determine how our search might have failed to see the SRIA. This led us to revise our search for some additives—for example, expanding our search for steviol glycoside to include rebaudioside A, and our search for polyglycerol polyricinoleate to include “pgpr.” After these modifications, the median percent of foods missed by our search decreased to 1.7%. A review of four remaining SRIAs for which we missed >5% of OFF-identified foods showed that our search correctly excluded the food >96% of the time. For example, a review of all instances showed that our search was correct in 242 (98%) of 247 instances for blue 1, and 372 (96%) of 387 instances for yellow 6. Examples of when our search incorrectly excluded the food were typographical errors (“blue !” instead of blue 1, or “yellow 5 and 36” instead of yellow 5 and 6), or instances in which an E number was listed without the letter “E” immediately preceding it.

### Analysis

For each food, we determined the total number of SRIAs (0–64) and the number of different types of SRIAs (0–4, i.e., additives used for texture, flavor, sweetness, and appearance). We also calculated proportions with at least one SRIA, each different type of SRIA, at least three SRIAs, and at least three of the four types of SRIAs, overall and by food group. The last two (3+ SRIAs, 3+ types of SRIAs) were arbitrarily selected as indicators of higher degree of ultra-processing.

## Results

**Figure 2:**
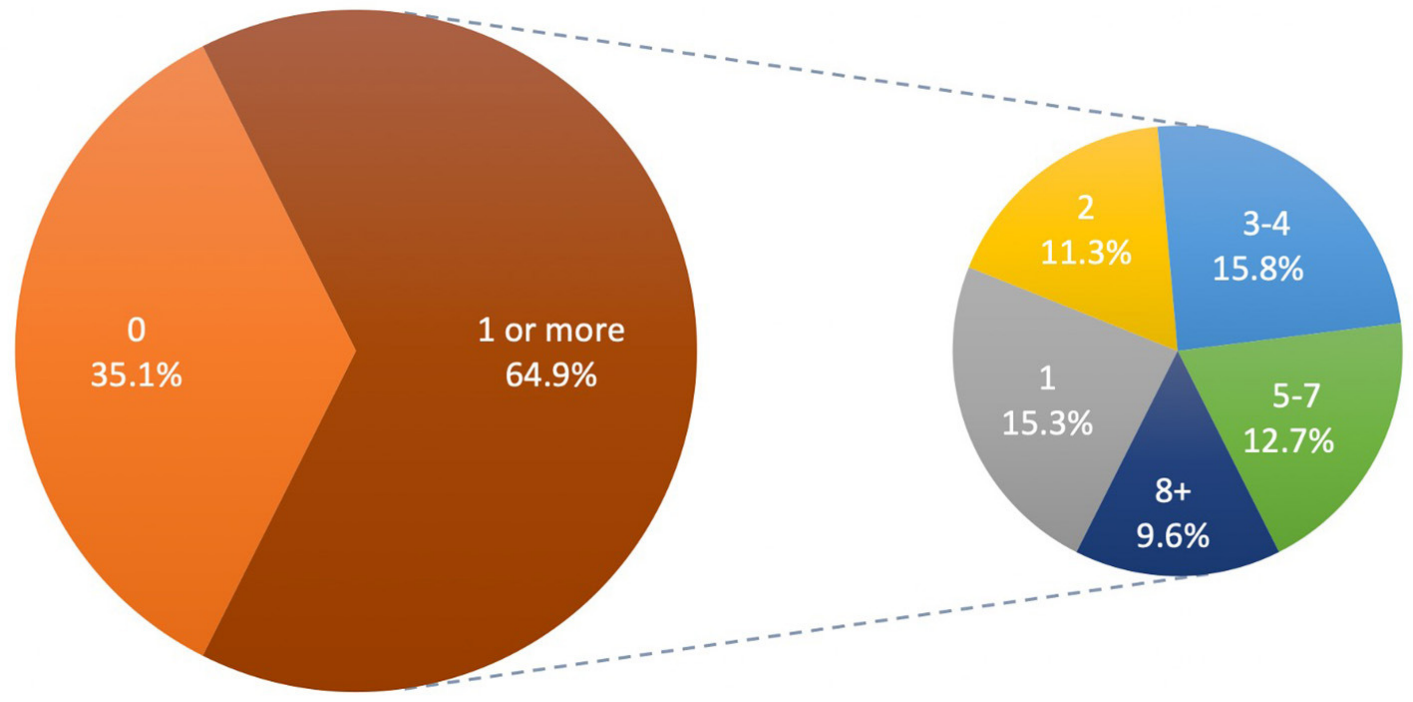
Proportions of foods in the Branded Food Products Database (*n* = 241,688) with one or more sensory-related industrial additives (SRIAs).

The percentages of foods with SRIAs are shown in Table 3 and Figure 2. Of 241,688 foods in the BFPD, almost two-thirds (64.9%) contained at least one SRIA from our list of 64 (Figure 2). Of these, over half (58.9%) had three or more SRIAs (Figure 2). Overall, the most prevalent SRIAs were for texture, which were present in 45.1% of all foods, followed by flavor (42.2%), sweetness (38.4%), then appearance (19.8%) (Table 3). Over a quarter (26.5%) of all foods contained at least three of the four SRIA types investigated (Table 3).

**Table 3:** Prevalence of sensory-related industrial additives (SRIA) and types of SRIA in food items in the Branded Food Products Database (*n* = 241,688).

	***N***	**%**
Any SRIAs	156,738	64.9
**Number of SRIAs**		
1	37,060	15.3
2	27,430	11.4
3–4	38,305	15.8
5–7	30,637	12.7
≥8	23,306	9.6
**Any SRIA related to**		
Texture	108,907	45.1
Flavor^a^	101,969	42.2
Sweetness	92,887	38.4
Appearance	47,941	19.8
**Number of SRIA types**		
1	48,473	20.1
2	44,134	18.3
3	41,561	17.2
4	22,570	9.3

a *Excludes sweetness*.

Among foods with information on food group, sweets (89.5%), non-alcoholic beverages (84.9%), and ready-to-eat (RTE) foods (82.0%) were the most likely to contain SRIAs (Table 4). Over half of the items in these categories contained three or more SRIAs. In contrast, 17.9% of items in the vegetable category contained SRIAs, and 8.2% contained three or more. Sweets (50.4%), non-alcoholic beverages (45.7%), and RTE foods (37.2%) were also the most likely to contain at least three of the four SRIA types, compared with <10% of fruits/juices, nuts, cheese, and vegetables.

**Table 4:** Prevalence (%) of sensory-related industrial additives (SRIA) in food items in the Branded Food Products Database, by food group (*n* = 224,098).

	**≥1 SRIA^**a**^**	**≥3 SRIA^**a**^**	**Texture**	**Flavor**	**Sweetness**	**Appearance**	**≥3 SRIA types^**b**^**
Sweets	**89.5**	**68.1**	**72.3**	**66.2**	**53.5**	42.2	**50.4**
Beverages	**84.9**	**57.7**	41.1	**71.6**	**63.6**	36.4	45.7
Ready-to-eat foods	**82.0**	**53.3**	**64.6**	**52.8**	**54.0**	20.8	37.2
Water	**70.0**	26.0	9.4	67.1	36.8	9.6	12.0
Meats	**66.0**	19.0	25.0	34.2	46.3	7.5	11.5
Milk/cream/yogurt	**65.1**	43.2	**56.0**	51.2	34.1	8.7	29.0
Dressings/condiments	**54.8**	25.4	38.0	32.6	26.7	15.6	18.2
Salty snacks	**56.7**	29.6	34.3	36.0	36.8	13.4	20.5
Fruits and juices	**55.2**	13.9	7.6	31.5	45.3	9.2	8.0
Coffee and tea	**53.7**	16.0	9.7	43.4	28.8	12.3	10.1
Grains	**52.0**	28.0	40.7	22.7	24.8	9.6	12.6
Fish	45.5	16.3	39.8	15.5	16.2	4.3	10.1
Nuts	42.4	5.4	36.0	7.8	13.7	0.3	1.3
Cheese	40.5	15.7	37.8	12.0	8.2	4.8	6.7
Vegetables	17.9	8.2	11.2	12.7	8.3	3.5	5.9

*Boldface indicates prevalence ≥50%*.

a *Number of individual SRIAs, with a possible maximum of 64*.

b *Number of types of SRIAs, with a possible maximum of four (texture, flavor, sweetness, appearance)*.

Prevalence of each of the four types of SRIAs and the specific SRIAs occurring in at least 10% of foods are shown in Supplementary Tables 2–5. Sweets were the most likely to contain texture related SRIAs, with 72.3% containing at least one (Table 4). The most common texture related SRIA used in sweets was lecithin, present in almost half of all products (Supplementary Table 2). Over half of RTE and milk products contained texture related SRIAs, with modified starches being the most common in RTE products, and carrageenan and modified starches the most common in milk products. The prevalence of texture related SRIAs was <10% for coffee and tea, water, and fruits and juices.

Flavor-related SRIAs were most prevalent in non-alcoholic beverages (71.6%), water products (67.1%), and sweets (66.2%). Natural flavors were by far the most common flavor related SRIA across food categories (Supplementary Table 3). Sweeteners were also the most prevalent in non-alcoholic beverages (63.6%), with high fructose corn syrup being the most common (Supplementary Table 4). Appearance-related SRIAs were present in <10% of foods for nine of the 15 food categories (Supplementary Table 5). However, they occurred in 42.2% of sweets/desserts, with the most common SRIAs being FD&C dyes.

## Discussion

### Summary and Discussion of Possible Mechanisms

Primary findings from this analysis were that most foods in the BFPD contained at least one SRIA, and over a third of foods had at least three. More than 80% of sweets, non-alcoholic beverages, and RTEs contained SRIAs. Additionally, more than a quarter of all food items in the BFPD had at least three of the four different categories of SRIAs examined, with texture related additives being the most common. Among sweets, 20% had all four categories.

Our analysis also points to specific SRIAs that appear the most frequently across food groups. For example, SRIAs that occurred the most frequently in sweets included lecithin, natural and artificial flavors, modified starches, mono/diglycerides, and dextrose. In RTE foods, frequently occurring SRIAs were natural flavors, modified food starches, xanthan gum, dextrose, and maltodextrin.

Industrial additives are frequently mentioned as a concern in consuming UPFs. A primary mechanism linking UPFs to disease risk is through enhanced sensory qualities that lead to overconsumption (18, 19). In a study conducted by Hall et al. (18), 20 adults were randomized to receive either ultra-processed or unprocessed diets for two weeks, followed by the alternate diet for two weeks. The two diets were matched for energy density and energy, macronutrient, fiber, sugar, and sodium content, and participants were instructed to consume as much or as little as desired. Hall et al. found that while participants lost weight during the unprocessed diet, they had greater energy intake and gained weight during the ultra-processed diet; this occurred despite no significant difference in reported palatability of the meals.

Small and DiFeliceantonio (19) suggest that additives in UPFs might co-opt existing pathways in which metabolic signals after consuming a food are conveyed from the gastrointestinal system to the brain. There, a rise in dopamine, part of the reward circuit response, reinforces the value of consuming that food, producing a reward response disproportionate to the food's caloric or nutrient content and thereby encouraging overconsumption. Notably, neural processing of these reinforcing signals appears to be independent of conscious perceptions about food, such as ratings of food liking or sensory pleasure. As a second possible mechanism, Fazzino et al. (28) suggest that foods with “multiple palatability-inducing ingredients” might weaken the sensory-specific satiety response, resulting in delayed signals for eating cessation and leading again to overconsumption (29). The extent to which these mechanisms are due to the presence of SRIAs, potentially combined with their relatively higher sodium and sugar content (30), has also yet to be explored, with implications for identification of specific additives of potential concern and product reformulation.

Worth noting is the possibility of product reformulation to *reduce* overconsumption. A recent pooled analysis found that energy intake rates are higher with consumption of UPF compared with less processed foods (31). In addition to suggesting a mechanism by which UPF might increase consumption independent of their SRIA content, the finding also suggests the possibility of reformulating UPFs to reduce intake rates—for example, by altering their form or texture (31, 32), taking advantage of technologies made possible with “ultra-processing”.

### Comparison With Related Studies

Baldridge et al. (25) classified >230,000 food and beverage products in Label Insight's Open Data database into 59 food categories, mapping each to NOVA categories representing levels of processing. Overall, they found that 70.9% of the 230,156 food and beverage products were ultra-processed. Ultra-processed food products comprised over 90% of convenience foods, sauces/dressings, bread/bakery products, and 100% of snack foods and confectionery. The discrepancy in results between the Baldridge et al.'s and our study is most likely because Baldridge et al. assigned entire categories of food to NOVA categories. In contrast, our estimates are based on actual ingredients listed. It is worth noting, however, that our findings are broadly similar: a significant majority of packaged food and beverage products in the US are “ultra-processed” according to the NOVA framework, including most sweets, beverages, convenience foods, meat, dairy, sauces/dressings, and snacks.

Dunford et al. (33) used a similar approach to our study—compiling a list of terms to search for in ingredient lists—to determine the proportion of branded food and beverage products containing non-nutritive sweeteners, using data from Label Insight's Open Data initiative. They found that the percent of products containing non-nutritive sweeteners in the US was 4.37%, close to our estimate of 4.29% (not shown). Dunford et al. also found that ~30–40% of soda, sports, and water drinks contained non-nutritive sweeteners, comparable with our estimates for beverages and waters. These findings indicate that a strategy of searching for additives of interest in ingredient lists can produce similar results, even when exact search terms vary. Of note, applying the same approach to Nielsen Homescan Consumer Panel data, Dunford et al. (34) found substantial changes in the prevalence of households purchasing non-nutritive sweeteners over time and differences by race/ethnicity, suggesting the additional insight gained from examining purchase data.

Chazelas et al. (21) examined additives in over 126,000 food and beverage products in the French market available in OFF. As described above in our Methods section, OFF searches parsed text in ingredient lists for specific additives (e.g., whey, invert sugar) or classes of additives (e.g., sequestrants, glazing agents) that are indicative of ultra-processed foods. They found that 53.8% of foods in the French packaged food supply contained at least one additive, with the highest proportions occurring in beverages, sweets, and convenience foods. Although Chazelas et al. did not limit their search to sensory related additives but included other categories such as preservatives, their estimate is lower than our estimate of 63.8%. The lower prevalence may be due to differences in the packaged food supply between France and the US, or differences in search procedures. Consistent with our findings, however, texture-related additives, such as lecithins, modified starches, and xanthan gum, were among the most frequently occurring additives in ingredient lists.

Batada et al. (35) estimated the prevalence of artificial food colors in 810 food and beverage products marketed toward children in one major supermarket in North Carolina. They found that 43.2% of products contained artificial food colors, with the most common being red 40 (29.8%), blue 1 (24.2%), yellow 5 (20.5), and yellow 6 (19.5%). Product categories with the highest percentages of artificial food colors were candies (96.3%), fruit-flavored snacks (94.7%), drink mixes/powders (89.7%), frozen breakfasts (85.7%), and toaster pastries (66.7%). The substantially higher percentages found by Batada et al. indicate the importance of appearance related SRIAs in marketing to children. Batada et al. (35) selected products only if they displayed a cartoon character or “bright and bubbly, child-friendly lettering” on the front of the package; advertised a child-oriented prize or incentive; and/or were thought to be a traditional children's item (e.g., fruit-flavored snacks).

### Strengths and Limitations

A limitation of this study is that it did not include all possible SRIAs due to the labor-intensive nature of searching ingredient lists. While we focused on only 64 SRIAs, E numbers exist for 41 colors, 19 sweeteners, 63 emulsifiers, stabilizers, thickeners, and gelling agents, and over 150 other additives with potentially relevant functions related to texture, flavor, and appearance (36). We chose to focus on the additives that appeared most indicative of industrial food production, and not typically used in domestic or culinary settings. However, our method is expandable; our shared program may be revised to include other SRIAs as deemed appropriate, and to be applied to other databases, including more updated versions of the BFPD, or even purchase data if linked to ingredient lists.

The prevalence of SRIAs may be underestimated because of incomplete ingredient lists, as well as variability in how the same SRIA can be written into ingredient lists, despite specific guidance to industry for food labeling (37). In addition, the BFPD does not represent all food products in the US marketplace. However, as the largest such analysis with over 240,000 items, our study offers a meaningful characterization of the US retail packaged food supply. Our results are relatively consistent with findings using other methods to identify additives or ultra-processed foods. Taken together, these studies collectively demonstrate the ubiquity of SRIAs in packaged food products, and most particularly in sweets, beverages, and RTE foods.

Although individual SRIAs have been linked to adverse health effects (17, 38, 39), estimating dosage or amounts of specific additives ingested by consumers was outside the scope of this study. However, our work is intended to build on previous studies on ultra-processed foods that treat ultra-processing as a binary characteristic. A binary classification system for processed foods is a potentially simple basis for developing guidelines for consumers and policymakers. But consumers may find some utility in distinguishing between UPFs containing one industrial sweetener from UPFs containing SRIAs that alter texture, flavor, and appearance. In research, distinguishing among foods that are more vs. less ultra-processed based on the number of SRIAs in a food offers a strategy for examining gradients in risk with degree of this aspect of ultra-processing. Considering the purpose of the additive is also potentially important. For example, Drewnowski (40) noted that >90% of plant-based alternatives to dairy milk contain industrial ingredients, using a similar search strategy as the one used here, but also including vitamins, minerals, and preservatives. Whether risk differs according to the number and types of additives in a food is a question that has not been addressed in previous research. Our approach offers a relatively straightforward way to quantify degree of one aspect of ultra-processing, using the presence of SRIAs as an indicator of the sensory qualities of a food.

Strengths of this study include its use of a large, publicly available database, and the development of open-source code meant to serve as a basis for other work. In addition, given the impracticality of recommending reduced intake of as broad and heterogeneous a class of foods as UPFs, quantifying the presence of SRIAs is a novel concept with implications for research exploring more specific mechanisms by which UPFs might affect health (41), and for developing actionable recommendations with respect to both consumer choice and industry product reformulation.

### Future Research

Our findings suggest three directions for future research. First, comparing this method with existing frameworks to classify UPFs will be useful both to quantify level of agreement across methods and to assess their relative usefulness in predicting disease risk. Second, a detailed description of correlations between SRIA occurrence and sodium, sugar, and fat content will help address whether and how SRIA and nutrient effects on disease risk might be disentangled. Third, examining the presence of SRIAs in purchase and intake data will provide necessary information on the level at which the US population is buying and consuming foods with SRIAs.

## Conclusion

In summary, our examination of the largest available database of packaged foods in the US confirms the ubiquity of SRIAs, especially in sweets, beverages, and RTE foods, but in all food groups to some extent. Quantifying the presence of SRIAs in foods offers a new approach to examine the aggregate effects of SRIAs on health outcomes, and to examine the assumption that UPFs increase disease risk through the presence of SRIAs and through effects on sensory qualities.

## Data Availability Statement

Publicly available datasets were analyzed in this study. This data can be found here: Branded Food Products Database https://fdc.nal.usda.gov; Open Food Facts https://us.openfoodfacts.org.

## Author Contributions

MT conceptualized the project, led the data analyses, contributed to data interpretation, and drafted the manuscript. CG assisted in selection of additives to include, developed the initial coding to search for text, investigated and edited ingredient text data prior to searching, conducted the comparisons with Open Food Facts, and contributed to data analyses and interpretation. AA assisted in selection of additives to include, developed the final coding to correct and search for text, reviewed output for errors and oversights, and contributed to data analyses and interpretation. SA provided input on sweeteners to include in searches. AN contributed to conceptualization and drafting the manuscript. All authors contributed to the article and approved the submitted version.

## Funding

This research was generously supported in part by the William and Linda Frost Fund in the Cal Poly College of Science and Mathematics.

## Conflict of Interest

The authors declare that the research was conducted in the absence of any commercial or financial relationships that could be construed as a potential conflict of interest.

## Publisher's Note

All claims expressed in this article are solely those of the authors and do not necessarily represent those of their affiliated organizations, or those of the publisher, the editors and the reviewers. Any product that may be evaluated in this article, or claim that may be made by its manufacturer, is not guaranteed or endorsed by the publisher.
