# Supplementary material for: Sensory-Related Industrial Additives in the US Packaged Food Supply
Source: Front Nutr. 2022 Jan 13;8:762814. doi: 10.3389/fnut.2021.762814 (PMC8792784; doi:10.3389/fnut.2021.762814)
Supplement: Supplementary file 1 [file Table_1.docx]

Supplementary Table 1. Examples of variability in appearance of selected additives, and search terms used.

| Additive | Examples of different appearance in ingredient lists | Search strategy |
| --- | --- | --- |
| maltodextrin | maltodextrine  maltodextrins  maltodextin  maltodextrin  maltodextrain  **matodextrin**  **malodextrin**  **maltdextrin**  maltodestrin  **meltodextrin**  maltodectrin  maltodext  maltodextain  **amltodextrin** | - Corrected spelling of boldfaced terms - Searched for ‘maltod’ |
| FD&C blue no. 1 | ble 1  blu 1  blue1  bluf 1  blur 1  blues 1  bule 1  blue 1  blue no 1 | - Corrected misspellings of ‘blue’ - Replaced ‘no.’ and ‘#’ with no - Searched for   - blue 1   - blue no 1   - E133   - brilliant blue |
| xanthan gum | xantham gum  xanthum gum  xantahn gum  xanthium gum  xathan gum  xanthangum  xantan gum  xhantan gum | - Excluded astaxanthin and canthaxanthin from search - Searched for   - xant   - E415 |

Supplementary Table 2. Prevalence of texture-related sensory-related industrial additives (SRIAs) by food category sorted according to prevalence, and SRIAs present in >10% of items.

|  | % | SRIAs present in >10% of food items | % |
| --- | --- | --- | --- |
| 1. sweets | 72.3 | lecithin | 48.0 |
|  |  | modified starch | 24.2 |
|  |  | mono/di-glycerides | 22.5 |
|  |  | hydrogenated oils | 14.2 |
|  |  | phosphates | 10.9 |
|  |  | carrageenan | 10.9 |
|  |  | Locust carob bean gum | 10.6 |
|  |  | polysorbates | 10.0 |
|  |  | cellulose | 10.0 |
| 2. ready-to-eat foods | 64.6 | modified food starch | 37.5 |
|  |  | xanthan gum | 23.0 |
|  |  | lecithin | 16.1 |
|  |  | phosphates | 12.9 |
|  |  | mono/di-glycerides | 11.2 |
|  |  | guar gum | 10.8 |
|  |  | cellulose | 10.2 |
| 3. milk/cream/yogurt | 56.0 | carrageenan | 22.9 |
|  |  | modified food starch | 22.0 |
|  |  | locust carob bean gum | 16.2 |
| 4. beverages | 41.1 | acacia gum | 14.0 |
|  |  | lecithin | 11.4 |
| 5. grains | 40.7 | mono/di-glycerides | 20.3 |
|  |  | lecithin | 16.8 |
| 6. fish | 39.8 | phosphates | 30.1 |
|  |  | modified food starch | 14.8 |
| 7. dressings/condiments | 38.0 | xanthan gum | 22.5 |
|  |  | modified food starch | 13.6 |
| 8. cheese | 37.8 | cellulose | 15.5 |
|  |  | guar gum | 14.0 |
|  |  | xanthan gum | 11.4 |
|  |  | locust carob bean gum | 11.0 |
| 9. nuts | 36.0 | hydrogenated oils | 23.6 |
|  |  | lecithin | 11.8 |
| 10. salty snacks | 34.3 | lecithin | 23.9 |
| 11. meats | 25.0 | modified food starch | 15.6 |
| 12. vegetables | 11.2 |  |  |
| 13. coffee and tea | 9.7 |  |  |
| 14. water | 9.4 |  |  |
| 15. fruits and juices | 7.6 |  |  |

Supplementary Table 3. Prevalence of flavor-related sensory-related industrial additives (SRIAs), excluding sweetness, by food category sorted according to prevalence, and SRIAs present in >10% of items.

|  | % | SRIAs present in >10% of food items | % |
| --- | --- | --- | --- |
| 1. beverages | 71.6 | natural flavor | 66.2 |
|  |  | artificial flavor | 28.7 |
| 2. water | 67.1 | natural flavor | 66.8 |
| 3. sweets | 66.2 | natural flavor | 48.9 |
|  |  | artificial flavor | 48.2 |
| 4. ready-to-eat foods | 52.8 | natural flavor | 46.3 |
|  |  | hydrolyzed soy/vegetable protein | 10.8 |
| 5. milk/cream/yogurt | 51.2 | natural flavor | 48.1 |
|  |  | artificial flavor | 13.5 |
| 6. coffee and tea | 43.4 | natural flavor | 42.6 |
| 7. salty snacks | 36.0 | natural flavor | 30.6 |
|  |  | artificial flavor | 10.4 |
| 8. meats | 34.1 | natural flavor | 30.8 |
| 9. dressings/condiments | 32.6 | natural flavor | 29.9 |
| 10. fruits and juices | 31.5 | natural flavor | 31.1 |
| 11. grains | 22.7 | natural flavor | 20.5 |
| 12. fish | 15.5 | natural flavor | 13.9 |
| 13. vegetables | 12.7 | natural flavor | 11.8 |
| 14. cheese | 12.0 | natural flavor | 11.6 |
| 15. nuts | 7.8 |  |  |

Supplementary Table 4. Prevalence of sweetener-related sensory-related industrial additives (SRIAs) by food category sorted according to prevalence, and SRIAs present in >10% of items.

|  | % | SRIAs present in >10% of food items | % |
| --- | --- | --- | --- |
| 1. beverages | 63.6 | high fructose corn syrup | 18.3 |
|  |  | sucralose | 14.3 |
|  |  | acesulfame | 15.3 |
|  |  | maltodextrin | 13.0 |
|  |  | stevia | 10.0 |
| 2. ready-to-eat foods | 54.0 | dextrose | 27.2 |
|  |  | maltodextrin | 23.3 |
|  |  | fruit juice concentrate | 10.2 |
| 3. sweets | 53.5 | dextrose | 20.9 |
|  |  | high fructose corn syrup | 14.6 |
| 4. meats | 46.3 | dextrose | 37.9 |
| 5. fruits and juices | 45.3 | fruit juice concentrate | 32.5 |
|  |  | high fructose corn syrup | 10.7 |
| 6. water | 36.8 | sucralose | 16.5 |
|  |  | acesulfame | 12.0 |
| 7. salty snacks | 36.8 | maltodextrin | 16.1 |
| 8. milk/cream/yogurt | 34.1 | fruit juice concentrate | 13.8 |
| 9. coffee and tea | 28.8 |  |  |
| 10. dressings/condiments | 26.7 |  |  |
| 11. grains | 24.8 |  |  |
| 12. fish | 16.2 |  |  |
| 13. nuts | 13.7 |  |  |
| 14. vegetables | 8.3 |  |  |
| 15. cheese | 8.2 |  |  |

Supplementary Table 5. Prevalence of appearance-related sensory-related industrial additives (SRIAs) by food category sorted according to prevalence, and SRIAs present in >10% of items.

|  | % | SRIAs present in >10% of food items | % |
| --- | --- | --- | --- |
| 1. sweets | 42.2 | FD&C red no.40 | 25.5 |
|  |  | FD&C yellow no.5 | 21.8 |
|  |  | FD&C blue no.1 | 21.0 |
|  |  | FD&C yellow no.6 | 16.1 |
|  |  | carnauba wax | 10.8 |
|  |  | titanium dioxide | 10.2 |
| 2. beverages | 36.4 | caramel color | 15.6 |
| 3. ready-to-eat foods | 20.8 | caramel color | 15.6 |
| 4. dressings/condiments | 15.6 |  |  |
| 5. salty snacks | 13.4 |  |  |
| 6. coffee and tea | 12.3 | caramel color | 10.7 |
| 7. grains | 9.6 |  |  |
| 8. water | 9.6 |  |  |
| 9. fruits and juices | 9.2 |  |  |
| 10. milk/cream/yogurt | 8.7 |  |  |
| 11. meats | 7.5 |  |  |
| 12. cheese | 4.8 |  |  |
| 13. fish | 4.3 |  |  |
| 14. vegetables | 3.5 |  |  |
| 15. nuts | 0.3 |  |  |
