# Supplementary material for: Sensory-Related Industrial Additives in the US Packaged Food Supply
Source: Front Nutr. 2022 Jan 13;8:762814. doi: 10.3389/fnut.2021.762814 (PMC8792784; doi:10.3389/fnut.2021.762814)
Supplement: Supplementary file 2 [file Data_Sheet_1.DOCX]

* THIS PROGRAM SEARCHES THE BRANDED FOOD PRODUCTS DATABASE

FOR 64 SENSORY-RELATED INDUSTRIAL ADDITIVES (SRIAS).

* INPUT DATASET IS DERIVED FROM THE DEC 2019 VERSION OF BFPD

BUT CAN BE REVISED TO MORE UPDATED VERSIONS.

* SOME PRE-PROCESSING OF THE BFPD IS NECESSARY

BEFORE IT CAN BE USED HERE.

NOTE THAT THE BFPD MUST BE IMPORTED IN A WAY

THAT DOES NOT TRUNCATE CHARACTER FIELDS (INCL INGREDIENTS).

;

libname library 'X:\pathname';

* EXCLUDE DUPLICATE UPC CODES;

proc sort data=library.bfpd1912; by gtin_upc; run;

data nodups;

set library.bfpd1912;

by gtin_upc;

if first.gtin_upc; * n=294,238, excludes n=37,033 duplicates;

run;

* EXCLUDE IF CLEARLY ERRONEOUS UPC, OR CLEARLY MISSING INGREDIENTS;

data temp0; * n=291,708;

set nodups;

if gtin_upc ne 'GDSN';

if ingredients ne '';

if ingredients ne 'makes ab';

run;

* PRE-PROCESSING OF INGREDIENTS DATA;

data temp1;

set temp0;

ingredients=lowcase(ingredients); *CONVERT ALL TO LOWER-CASE;

ingredients=tranwrd(ingredients,'-',' '); *REPLACE SYMBOLS;

ingredients=tranwrd(ingredients,'#',' ');

ingredients=tranwrd(ingredients,'no.','no ');

ingredients=tranwrd(ingredients,'&',' and ');

* STANDARDIZE SPELLING;

ingredients=tranwrd(ingredients,'colour','color');

ingredients=tranwrd(ingredients,'flavour','flavor');

ingredients=compbl(ingredients); *REMOVE MULTIPLE BLANKS;

run;

* EXCLUDE DUPLICATES IDENTIFIED BY FOOD NAME/DESCRIPTION;

data temp1a;

set temp1;

length brand_descr $ 180;

brand_descr = cat(brand_owner,description);

run;

proc sort data=temp1a; by brand_descr; run;

data temp1b;

set temp1a;

by brand_descr;

if first.brand_descr; * n=241,688;

run;

*********************************************************************

* THIS SECTION BEGINS SEARCH FOR SRIAS *

*********************************************************************;

**************

* SWEETENERS *

**************;

* NUTRITIVE SWEETENERS:

HFCS, SORBITOL, MANNITOL, XYLITOL, ERYTHRITOL,

MALTITOL, AND POLYGLYCITOL SYRUP ;

data hfcs;

set temp1b;

* hfcs;

* CORRECT MISSPELLINGS;

ingredients=tranwrd(ingredients,'fluctose','fructose');

ingredients=tranwrd(ingredients,'froctose','fructose');

ingredients=tranwrd(ingredients,'frucose','fructose');

ingredients=tranwrd(ingredients,'frucrtose','fructose');

ingredients=tranwrd(ingredients,'fructise','fructose');

ingredients=tranwrd(ingredients,'fructoe','fructose');

ingredients=tranwrd(ingredients,'fructos','fructose');

ingredients=tranwrd(ingredients,'fructosez','fructose');

ingredients=tranwrd(ingredients,'fructrose','fructose');

ingredients=tranwrd(ingredients,'frugtose','fructose');

ingredients=tranwrd(ingredients,'frustose','fructose');

ingredients=tranwrd(ingredients,'frutcose','fructose');

ingredients=tranwrd(ingredients,'frutose','fructose');

ingredients=tranwrd(ingredients,'frutrose','fructose');

ingredients=tranwrd(ingredients,'highfructose','high fructose');

* THESE STATEMENTS SERVE TO EXCLUDE HIGH FRUCTOSE RICE SYRUP

AND HAVING NO HFCS FROM THE SEARCH;

ingredients=tranwrd(ingredients,'high fructose rice syrup','hf rice syrup');

ingredients=tranwrd(ingredients,'no high fructose','no hf');

* CREATE INDICATOR;

if (index(ingredients,'high fructose')>0) then highfructose=1;

else highfructose=0;

* sorbitol;

ingredients=tranwrd(ingredients,'sarbitol','sorbitol');

ingredients=tranwrd(ingredients,'sorbirtol','sorbitol');

ingredients=tranwrd(ingredients,'sorbital','sorbitol');

ingredients=tranwrd(ingredients,'sorbitil','sorbitol');

ingredients=tranwrd(ingredients,'sorbitiol','sorbitol');

ingredients=tranwrd(ingredients,'sorbltol','sorbitol');

if (index(ingredients,'sorbitol')>0)

or (index(ingredients,'e420')>0) then sorbitol=1;

else sorbitol=0;

* mannitol;

ingredients=tranwrd(ingredients,'maniitol','mannitol');

if (index(ingredients,'mannit')>0)

or (index(ingredients,'e421')>0) then mannitol=1;

else mannitol=0;

* xylitol;

ingredients=tranwrd(ingredients,'xyitol','xylitol');

if (index(ingredients,'xyli')>0)

or (index(ingredients,'e967')>0) then xylitol=1;

else xylitol=0;

* erythritol;

ingredients=tranwrd(ingredients,'erythetol','erythritol');

ingredients=tranwrd(ingredients,'erythitol','erythritol');

if (index(ingredients,'erythri')>0)

or (index(ingredients,'ritol')>0)

or (index(ingredients,'e968')>0) then erythritol=1;

else erythritol=0;

* maltitol;

ingredients=tranwrd(ingredients,'maliitol','maltitol');

ingredients=tranwrd(ingredients,'malitol','maltitol');

ingredients=tranwrd(ingredients,'malitotol','maltitol');

ingredients=tranwrd(ingredients,'maltiol','maltitol');

* EXCLUDE LACTITOL FROM SEARCH;

ingredients=tranwrd(ingredients,'lactitol','lac_itol');

if (index(ingredients,'maltit')>0)

or (index(ingredients,'titol')>0)

or (index(ingredients,'e965')>0) then maltitol=1;

else maltitol=0;

* polyglycitol syrup;

ingredients=tranwrd(ingredients,'polyglucitol','polyglycitol syrup');

ingredients=tranwrd(ingredients,'polyglycol syrup','polyglycitol syrup');

if (index(ingredients,'polyglyci')>0)

or (index(ingredients,'e964')>0) then polyglycitolsyrup=1;

else polyglycitolsyrup =0;

* cane juice;

ingredients=tranwrd(ingredients,'caner j','cane j');

if (index(ingredients,'cane j')>0) then canejuice=1;

else canejuice=0;

* corn syrup solids;

ingredients=tranwrd(ingredients,'solds','solids');

ingredients=tranwrd(ingredients,'solide','solids');

ingredients=tranwrd(ingredients,'solids‚','solids');

ingredients=tranwrd(ingredients,'syrp s','syrup solids');

ingredients=tranwrd(ingredients,'syrupsolids','syrup solids');

ingredients=tranwrd(ingredients,'sytup','syrup');

ingredients=tranwrd(ingredients,'powder, com','powder, corn');

if (index(ingredients,'syrup solid')>0) then cornsyrupsolids=1;

else cornsyrupsolids=0;

* dextrose;

ingredients=tranwrd(ingredients,'dectrose','dextrose');

ingredients=tranwrd(ingredients,'destrose','dextrose');

ingredients=tranwrd(ingredients,'desxtrose','dextrose');

ingredients=tranwrd(ingredients,'detrose','dextrose');

ingredients=tranwrd(ingredients,'dexdrose','dextrose');

ingredients=tranwrd(ingredients,'dexrose','dextrose');

ingredients=tranwrd(ingredients,'dexrtose','dextrose');

ingredients=tranwrd(ingredients,'dextorse','dextrose');

ingredients=tranwrd(ingredients,'dextose','dextrose');

ingredients=tranwrd(ingredients,'dextrise','dextrose');

ingredients=tranwrd(ingredients,'dextro','dextrose');

ingredients=tranwrd(ingredients,'dextroe','dextrose');

ingredients=tranwrd(ingredients,'dextronse','dextrose');

ingredients=tranwrd(ingredients,'dextrsoe','dextrose');

ingredients=tranwrd(ingredients,'dxtrose','dextrose');

ingredients=tranwrd(ingredients,'dxtrse','dextrose');

* EXCLUDE THESE;

ingredients=tranwrd(ingredients,'dextrosemonohydrate','dextrose_onohydrate');

ingredients=tranwrd(ingredients,'paltdextrose','poly_extrose');

ingredients=tranwrd(ingredients,'poldextrose','poly_extrose');

ingredients=tranwrd(ingredients,'ydextrose','y_extrose');

if (index(ingredients,'dextros')>0)

or (index(ingredients,'dex tros')>0) then dextrose=1;

else dextrose=0;

* rice syrup;

if (index(ingredients,'rice syrup')>0) then ricesyrup=1;

else ricesyrup=0;

* fructose

NOTE THAT FRUCTOSE IS COUNTED ONLY FOR FOODS NOT ALREADY

CONTAINING HF CORN OR RICE SYRUP;

if (highfructose=0) and (ricesyrup=0) then

do;

if (index(ingredients,'fructose')>0) then fructose=1;

else fructose=0;

end;

else fructose=0;

* fruit juice concentrate;

ingredients=tranwrd(ingredients,'centrate','concentrate');

ingredients=tranwrd(ingredients,'cncentrate','concentrate');

ingredients=tranwrd(ingredients,'cocentrate','concentrate');

ingredients=tranwrd(ingredients,'fruitcentrate','fruit concentrate');

if (index(ingredients,'juice con')>0) then fruitjuicecon=1;

else fruitjuicecon=0;

* glucose;

ingredients=tranwrd(ingredients,'glucosa','glucose');

ingredients=tranwrd(ingredients,'cluclose','glucose');

ingredients=tranwrd(ingredients,'glucese','glucose');

if (index(ingredients,'glucose')>0) then glucose=1;

else glucose=0;

* invert sugar;

ingredients=tranwrd(ingredients,'invertsugar','invert sugar');

ingredients=tranwrd(ingredients,'inverted','invert');

ingredients=tranwrd(ingredients,'invest sugar','invert sugar');

ingredients=tranwrd(ingredients,'invent','invert');

ingredients=tranwrd(ingredients,'invented','invert');

if (index(ingredients,'invert s')>0)

or (index(ingredients,'invert ca')>0) then invertsugar=1;

else invertsugar=0;

* maltodextrin;

ingredients=tranwrd(ingredients,'matodextrin','maltodextrin');

ingredients=tranwrd(ingredients,'malodextrin','maltodextrin');

ingredients=tranwrd(ingredients,'maltdextrin','maltodextrin');

ingredients=tranwrd(ingredients,'meltodextrin','maltodextrin');

ingredients=tranwrd(ingredients,'amltodextrin','maltodextrin');

ingredients=tranwrd(ingredients,'maltidextrin','maltodextrin');

ingredients=tranwrd(ingredients,'mlatodextrin','maltodextrin');

ingredients=tranwrd(ingredients,'maitodextrin','maltodextrin');

ingredients=tranwrd(ingredients,'maliodextrin','maltodextrin');

ingredients=tranwrd(ingredients,'malrodextrin','maltodextrin');

ingredients=tranwrd(ingredients,'maltrdextrin','maltodextrin');

ingredients=tranwrd(ingredients,'maltrodextribs','maltodextrin');

ingredients=tranwrd(ingredients,'malttodextrine','maltodextrin');

ingredients=tranwrd(ingredients,'malttodextrin','maltodextrin');

ingredients=tranwrd(ingredients,'matlodextrin','maltodextrin');

ingredients=tranwrd(ingredients,'mattlodextrin','maltodextrin');

ingredients=tranwrd(ingredients,'matitodextrin','maltodextrin');

ingredients=tranwrd(ingredients,'matodextrin','maltodextrin');

ingredients=tranwrd(ingredients,'matodexterin','maltodextrin');

ingredients=tranwrd(ingredients,'mnaltodextrin','maltodextrin');

ingredients=tranwrd(ingredients,'moltodextrin','maltodextrin');

ingredients=tranwrd(ingredients,'maltosextrin','maltodextrin');

if (index(ingredients,'maltod')>0) then maltodextrin=1;

else maltodextrin=0;

* dextrin;

* EXCLUDE THESE;

ingredients=tranwrd(ingredients,'oligodextrin','oligo_extrin');

ingredients=tranwrd(ingredients,'oliodextrin','olio_extrin');

ingredients=tranwrd(ingredients,'polydextrin','poly_extrin');

ingredients=tranwrd(ingredients,'maltodextrin','malt_extrin');

* The maltodextrin statement above must come after the search

for maltodextrin;

if (index(ingredients,'dextrin')>0) then dextrin=1;

else dextrin=0;

* maltose;

ingredients=tranwrd(ingredients,'maltode','maltose');

if (index(ingredients,'maltose')>0) then maltose=1;

else maltose=0;

* maltol;

if (index(ingredients,'maltol')>0) then maltol=1;

else maltol=0;

* refiners syrup;

if (index(ingredients,"refiner's sy")>0)

or (index(ingredients,'refiners sy')>0)

or (index(ingredients,'refinery sy')>0)

or (index(ingredients,'refines sy')>0) then refinerssyrup=1;

else refinerssyrup=0;

* CREATE INDICATORS FOR # NUTRITIVE SWEETENERS (0-20),

AND BINARY VARIABLE (1=YES, 0=NO);

ns20 = highfructose + sorbitol + mannitol + xylitol + erythritol +

maltitol + polyglycitolsyrup + canejuice + cornsyrupsolids +

dextrose + glucose + maltodextrin + maltol + ricesyrup + dextrin +

maltose + fructose + fruitjuicecon + invertsugar + refinerssyrup;

if ns20>0 then ns=1; else ns=0;

* NON-NUTRITIVE SWEETENERS:

Acesulfame potassium, Aspartame, Neotame,

Saccharin, Stevia, Sucralose;

*sucralose;

ingredients=tranwrd(ingredients,'sucarlose','sucralose');

ingredients=tranwrd(ingredients,'succlarose','sucralose');

ingredients=tranwrd(ingredients,'sucralaose','sucralose');

ingredients=tranwrd(ingredients,'sucralsoe','sucralose');

ingredients=tranwrd(ingredients,'sucrolose','sucralose');

ingredients=tranwrd(ingredients,'sucrolse','sucralose');

ingredients=tranwrd(ingredients,'sucrslose','sucralose');

ingredients=tranwrd(ingredients,'sucrulose','sucralose');

ingredients=tranwrd(ingredients,'sucraiose','sucralose');

ingredients=tranwrd(ingredients,'sugralose','sucralose');

ingredients=tranwrd(ingredients,'sugarlose','sucralose');

ingredients=tranwrd(ingredients,'suralose','sucralose');

ingredients=tranwrd(ingredients,'surcalose','sucralose');

ingredients=tranwrd(ingredients,'surcralose','sucralose');

ingredients=tranwrd(ingredients,'sacralose','sucralose');

ingredients=tranwrd(ingredients,'socralose','sucralose');

if (index(ingredients,'sucralose')>0)

or (index(ingredients,'e955')>0) then sucralose=1;

else sucralose=0;

* saccharin;

ingredients=tranwrd(ingredients,'sacchanrin','saccharin');

if (index(ingredients,'saccharin')>0)

or (index(ingredients,'e954')>0) then saccharin=1;

else saccharin=0;

* acesulfame;

ingredients=tranwrd(ingredients,'asesulfame','acesulfame');

ingredients=tranwrd(ingredients,'acsulfame','acesulfame');

ingredients=tranwrd(ingredients,'ascesulfame','acesulfame');

ingredients=tranwrd(ingredients,'acesfulfame','acesulfame');

ingredients=tranwrd(ingredients,'aceslfame','acesulfame');

ingredients=tranwrd(ingredients,'asulfame','acesulfame');

if (index(ingredients,'acesu')>0)

or (index(ingredients,'e950')>0) then acesu=1;

else acesu=0;

* aspartame;

ingredients=tranwrd(ingredients,'aspertame','aspartame');

ingredients=tranwrd(ingredients,'apsartame','aspartame');

ingredients=tranwrd(ingredients,'asparatame','aspartame');

ingredients=tranwrd(ingredients,'aspatrame','aspartame');

* EXCLUDE THESE;

ingredients=tranwrd(ingredients,'aspartic','aspar_ic');

ingredients=tranwrd(ingredients,'aspartate','aspar_ate');

if (index(ingredients,'aspart')>0)

or (index(ingredients,'e951')>0) then aspart=1;

else aspart=0;

* neotame;

ingredients=tranwrd(ingredients,'neo tame','neotame');

if (index(ingredients,'neotame')>0)

or (index(ingredients,'e961')>0) then neotame=1;

else neotame=0;

* stevia;

* EXCLUDE THIS;

ingredients=tranwrd(ingredients,'steve','ste_e');

if (index(ingredients,'stev')>0)

or (index(ingredients,'e960')>0)

or (index(ingredients,'rebau')>0)

or (index(ingredients,'rebiana')>0) then stev=1;

else stev=0;

* CREATE INDICATORS FOR # NON-NUTRITIVE SWEETENERS (0-6),

AND BINARY VARIABLE (1=YES, 0=NO);

nns6 = Acesu + Aspart + Neotame + Saccharin + Stev + Sucralose;

if nns6>0 then nns=1; else nns=0;

* CREATE INDICATORS FOR TOTAL SWEETENERS (0-26),

AND BINARY VARIABLE (1=YES, 0=NO);

sweet26 = ns20 + nns6;

if sweet26>0 then sweet=1; else sweet=0;

run;

***********

* FLAVORS *

***********;

data artificialflavor;

set hfcs;

* artificial flavor;

ingredients=tranwrd(ingredients,'favoring','flavoring');

ingredients=tranwrd(ingredients,'favors','flavors');

ingredients=tranwrd(ingredients,'favour','flavor');

ingredients=tranwrd(ingredients,'favor,','flavor,');

ingredients=tranwrd(ingredients,'favor)','flavor)');

ingredients=tranwrd(ingredients,'favor.','flavor.');

ingredients=tranwrd(ingredients,'flvaor','flavor');

ingredients=tranwrd(ingredients,'flvor','flavor');

ingredients=tranwrd(ingredients,'flvor','flavor');

ingredients=tranwrd(ingredients,'flvr','flavor');

ingredients=tranwrd(ingredients,'flv','flavor');

ingredients=tranwrd(ingredients,'artificialflavor','artificial flavor');

ingredients=tranwrd(ingredients,'artificial favirs','artificial flavor');

ingredients=tranwrd(ingredients,'art flavor','artificial flavor');

ingredients=tranwrd(ingredients,'art. flavor','artificial flavor');

ingredients=tranwrd(ingredients,'art. i flavor','artificial flavor');

ingredients=tranwrd(ingredients,'artificial, flavor','artificial flavor');

* EXCLUDE IF INGREDIENTS SAY NO ARTIFICIAL FLAVORS;

ingredients=tranwrd(ingredients,'no artificial flav','no artif flav');

if (index(ingredients,'artificial flav')>0)

or (index(ingredients,'artificial butter')>0)

or (index(ingredients,'artificial vanilla')>0)

or (index(ingredients,'artificial and natural flav')>0)

then artificialflavor=1;

else artificialflavor=0;

* natural flavor;

ingredients=tranwrd(ingredients,'nature identical','natural');

ingredients=tranwrd(ingredients,'naturual','natural');

ingredients=tranwrd(ingredients,'natrual','natural');

ingredients=tranwrd(ingredients,'natr ','natural');

ingredients=tranwrd(ingredients,'natral','natural');

ingredients=tranwrd(ingredients,'natual','natural');

ingredients=tranwrd(ingredients,'natuarl','natural');

ingredients=tranwrd(ingredients,'natura ','natural');

ingredients=tranwrd(ingredients,'naturals','natural');

ingredients=tranwrd(ingredients,'naturl','natural');

ingredients=tranwrd(ingredients,'naturla','natural');

ingredients=tranwrd(ingredients,'naural','natural');

ingredients=tranwrd(ingredients,'nautral','natural');

ingredients=tranwrd(ingredients,'ntrl','natural');

ingredients=tranwrd(ingredients,'naturaland','natural and');

ingredients=tranwrd(ingredients,'nat.','nat');

ingredients=tranwrd(ingredients,'nat flav','natural flav');

ingredients=tranwrd(ingredients,'nat and art','natural and art');

ingredients=tranwrd(ingredients,'ntrl0art','natural and art');

ingredients=tranwrd(ingredients,'nature flavor','natural flavor');

ingredients=tranwrd(ingredients,'naturalflavor','natural flavor');

if (index(ingredients,'natural flav')>0)

or (index(ingredients,'natural and artificial flav')>0)

then naturalflavor=1;

else naturalflavor=0;

* disodium guanylate;

ingredients=tranwrd(ingredients,'guanylate','disodium guanylate');

* EXCLUDE THESE;

ingredients=tranwrd(ingredients,'succinylated','succi_ylated');

ingredients=tranwrd(ingredients,'guanabana','gua_abana');

ingredients=tranwrd(ingredients,'nicaraguan','Nicaragua_');

ingredients=tranwrd(ingredients,'guanosine','gua_osine');

ingredients=tranwrd(ingredients,'tieguanyin','tiegua_yin');

if (index(ingredients,'guan')>0)

or (index(ingredients,'nylat')>0)

or (index(ingredients,'e627')>0) then disodiumguanylate=1;

else disodiumguanylate=0;

* disodium inosinate;

ingredients=tranwrd(ingredients,'insoinate','inosinate');

ingredients=tranwrd(ingredients,'inosnate','inosinate');

ingredients=tranwrd(ingredients,'insonate','inosinate');

ingredients=tranwrd(ingredients,'inodinate','inosinate');

ingredients=tranwrd(ingredients,'inonate','inosinate');

ingredients=tranwrd(ingredients,'inoslnate','inosinate');

ingredients=tranwrd(ingredients,'isonate','inosinate');

ingredients=tranwrd(ingredients,'insoninate','inosinate');

* EXCLUDE THESE;

ingredients=tranwrd(ingredients,'aluminosi','alumino_si');

ingredients=tranwrd(ingredients,'askinosie','askinos_e');

if (index(ingredients,'inosi')>0)

or (index(ingredients,'sodium inos')>0)

or (index(ingredients,'e631')>0) then disodiuminosinate=1;

else disodiuminosinate=0;

* autolyzed yeast extract;

ingredients=tranwrd(ingredients,'autilyzed','autolyzed yeast extract');

ingredients=tranwrd(ingredients,'autholyzed','autolyzed yeast extract');

ingredients=tranwrd(ingredients,'zutolyzed','autolyzed yeast extract');

if (index(ingredients,'auto')>0) then autolyzedyeastextract=1;

else autolyzedyeastextract=0;

* hydrolyzed vegetable protein;

ingredients=tranwrd(ingredients,'hydrolysate','hydrolysate');

ingredients=tranwrd(ingredients,'hydrolysed','hydrolyzed');

ingredients=tranwrd(ingredients,'hydrolyzate','hydrolysate');

ingredients=tranwrd(ingredients,'hydrolyced','hydrolyzed');

ingredients=tranwrd(ingredients,'hydrolysedsoyprotein','hydrolyzed soy protein');

ingredients=tranwrd(ingredients,'hydrolyzes','hydrolyzed');

ingredients=tranwrd(ingredients,'hydrokyzrd','hydrolyzed');

ingredients=tranwrd(ingredients,'soyprotein','soy protein');

ingredients=tranwrd(ingredients,'hydroglyzed','hydrolyzed');

ingredients=tranwrd(ingredients,'hodrolyzed','hydrolyzed');

ingredients=tranwrd(ingredients,'htdrolyzed','hydrolyzed');

ingredients=tranwrd(ingredients,'hydolyzed','hydrolyzed');

ingredients=tranwrd(ingredients,'hydrelyzed','hydrolyzed');

ingredients=tranwrd(ingredients,'hyrdolyzed','hydrolyzed');

ingredients=tranwrd(ingredients,'hydrolized','hydrolyzed');

ingredients=tranwrd(ingredients,'lyzed','hydrolyzed');

ingredients=tranwrd(ingredients,'hydroylzed','hydrolyzed');

ingredients=tranwrd(ingredients,'hydrolzed','hydrolyzed');

ingredients=tranwrd(ingredients,'hyrolyzed','hydrolyzed');

ingredients=tranwrd(ingredients,'hydorlized','hydrolyzed');

ingredients=tranwrd(ingredients,'hydroliyzed','hydrolyzed');

ingredients=tranwrd(ingredients,'hydrolyzrd','hydrolyzed');

ingredients=tranwrd(ingredients,'soy protien','soy protein');

ingredients=tranwrd(ingredients,'cornsoy','corn');

* EXCLUDE THESE;

ingredients=tranwrd(ingredients,'hydrolyzed soybean oil','hydrolyzed so_bean oil');

ingredients=tranwrd(ingredients,'hydrolyzed corn starch','hydrolyzed co_n starch');

if (index(ingredients,'hydrolyzed soy')>0)

or (index(ingredients,'hydrolyzed vegetable prot')>0)

or (index(ingredients,'hydrolyzed corn')>0)

or (index(ingredients,'hydrolyzed wheat')>0)

or (index(ingredients,'hydrolyzed plant')>0)

or (index(ingredients,'soy protein hydrolysate')>0)

or (index(ingredients,'vegetable protein hydrolysate')>0)

or (index(ingredients,'corn protein hydrolysate')>0)

then hydrolyzedvegetableprotein=1;

else hydrolyzedvegetableprotein=0;

run;

data flavors;

set artificialflavor;

* CREATE INDICATORS FOR FLAVORS (0-6),

AND BINARY VARIABLE (1=YES, 0=NO);

flavor6 = artificialflavor + naturalflavor + disodiumguanylate +

autolyzedyeastextract + disodiuminosinate +

hydrolyzedvegetableprotein;

if flavor6>0 then flavor=1; else flavor=0;

run;

**************

* APPEARANCE *

**************;

data blue;

set flavors;

* blue 1 and 2;

ingredients=tranwrd(ingredients,'blue lake','blue');

ingredients=tranwrd(ingredients,'ble 1','blue 1');

ingredients=tranwrd(ingredients,'blu 1','blue 1');

ingredients=tranwrd(ingredients,'blue1','blue 1');

ingredients=tranwrd(ingredients,'blue2','blue 2');

ingredients=tranwrd(ingredients,'bluf 1','blue 1');

ingredients=tranwrd(ingredients,'blur 1','blue 1');

ingredients=tranwrd(ingredients,'blues 1','blue 1');

ingredients=tranwrd(ingredients,'bule 1','blue 1');

if (index(ingredients,'blue 1')>0)

or (index(ingredients,'blue no 1')>0)

or (index(ingredients,'e133')>0)

or (index(ingredients,'brilliant blue')>0) then blue1=1;

else blue1=0;

if (index(ingredients,'blue 2')>0)

or (index(ingredients,'blue no 2')>0)

or (index(ingredients,'e132')>0)

or (index(ingredients,'indigo carmine')>0) then blue2=1;

else blue2=0;

* green 3;

if (index(ingredients,'green 3')>0)

or (index(ingredients,'e143')>0)

or (index(ingredients,'fast green')>0) then green3=1;

else green3=0;

* red 3 and 40;

ingredients=tranwrd(ingredients,'red lake','red');

ingredients=tranwrd(ingredients,'fd&c ed 40','fd&c red 40');

ingredients=tranwrd(ingredients,'red3','red 3');

ingredients=tranwrd(ingredients,'red40','red 40');

ingredients=tranwrd(ingredients,'re 40','red 40');

ingredients=tranwrd(ingredients,'rede 40','red 40');

ingredients=tranwrd(ingredients,'redo 40','red 40');

ingredients=tranwrd(ingredients,'reed 40','red 40');

ingredients=tranwrd(ingredients,'no40','no 40');

if ((index(ingredients,'red 3')>0)

or (index(ingredients,'red no 3')>0))

or (index(ingredients,'reds 3')>0)

or (index(ingredients,'reds 40')>0)

or (index(ingredients,'e127')>0)

or (index(ingredients,'erythrosine')>0) then red3=1;

else red3=0;

if ((index(ingredients,'red 40')>0)

or (index(ingredients,'red no 40')>0))

or (index(ingredients,'reds 3')>0)

or (index(ingredients,'reds 40')>0)

or (index(ingredients,'e129')>0)

or (index(ingredients,'allura red')>0) then red40=1;

else red40=0;

* yellow 5 and 6;

ingredients=tranwrd(ingredients,'yellow lake','yellow');

ingredients=tranwrd(ingredients,'yellows lakes','yellows');

ingredients=tranwrd(ingredients,'yello0w','yellow');

ingredients=tranwrd(ingredients,'yellos','yellow');

ingredients=tranwrd(ingredients,'yellow5','yellow 5');

ingredients=tranwrd(ingredients,'yellow6','yellow 6');

ingredients=tranwrd(ingredients,'yellw','yellow');

ingredients=tranwrd(ingredients,'yelow n5','yellow 5');

ingredients=tranwrd(ingredients,'yelow','yellow');

ingredients=tranwrd(ingredients,'yellows 5.','yellow 5');

ingredients=tranwrd(ingredients,'yellows 5,','yellow 5');

ingredients=tranwrd(ingredients,'yellow no5','yellow 5');

ingredients=tranwrd(ingredients,'yellow no6','yellow 6');

ingredients=tranwrd(ingredients,'yllw','yellow');

if ((index(ingredients,'yellow 5')>0)

or (index(ingredients,'yellow no 5')>0))

or (index(ingredients,'yellows 5')>0)

or (index(ingredients,'yellows 6')>0)

or (index(ingredients,'e102')>0)

or (index(ingredients,'tartrazine')>0) then yellow5=1;

else yellow5=0;

if ((index(ingredients,'yellow 6')>0)

or (index(ingredients,'yellow no 6')>0))

or (index(ingredients,'yellows 5')>0)

or (index(ingredients,'yellows 6')>0)

or (index(ingredients,'e110')>0)

or (index(ingredients,'sunset yellow')>0)

or (index(ingredients,'yellow 5 and 6')>0) then yellow6=1;

else yellow6=0;

* artificial color;

ingredients=tranwrd(ingredients,'arificial','artificial');

ingredients=tranwrd(ingredients,'aritfical','artificial');

ingredients=tranwrd(ingredients,'aritficial','artificial');

ingredients=tranwrd(ingredients,'aritifical','artificial');

ingredients=tranwrd(ingredients,'aritificial','artificial');

ingredients=tranwrd(ingredients,'artfcl','artificial');

ingredients=tranwrd(ingredients,'artficial','artificial');

ingredients=tranwrd(ingredients,'articial','artificial');

ingredients=tranwrd(ingredients,'articifial','artificial');

ingredients=tranwrd(ingredients,'artifcial','artificial');

ingredients=tranwrd(ingredients,'artifial','artificial');

ingredients=tranwrd(ingredients,'artificail','artificial');

ingredients=tranwrd(ingredients,'artifical','artificial');

ingredients=tranwrd(ingredients,'artifice','artificial');

ingredients=tranwrd(ingredients,'artificl','artificial');

ingredients=tranwrd(ingredients,'artifiicial','artificial');

ingredients=tranwrd(ingredients,'artifiticial','artificial');

ingredients=tranwrd(ingredients,'atificial','artificial');

ingredients=tranwrd(ingredients,'artificially','artificial');

* above is not to correct misspelling but for ease of search;

ingredients=tranwrd(ingredients,'artifically','artificial');

ingredients=tranwrd(ingredients,'artificialcolor','artificial color');

ingredients=tranwrd(ingredients,'atfi, color','artificial color');

ingredients=tranwrd(ingredients,'colorantes artificiales','artificial color');

ingredients=tranwrd(ingredients,'artificia;','artificial');

* EXCLUDE IF INGREDIENTS SAY NO ARTIFICIAL COLORS;

ingredients=tranwrd(ingredients,'no artificial col','no artif col');

if (index(ingredients,'artificial col')>0)

or (index(ingredients,'artificial and natural col')>0)

then artificialcolor=1;

else artificialcolor=0;

* titanium dioxide;

ingredients=tranwrd(ingredients,'titamium','titanium dioxide');

ingredients=tranwrd(ingredients,'titanum','titanium dioxide');

ingredients=tranwrd(ingredients,'titatium','titanium dioxide');

ingredients=tranwrd(ingredients,'totitanium','titanium dioxide');

if (index(ingredients,'titani')>0)

or (index(ingredients,'e171')>0) then titaniumdioxide=1;

else titaniumdioxide=0;

* caramel color;

ingredients=tranwrd(ingredients,'capamel','caramel');

ingredients=tranwrd(ingredients,'caramal','caramel');

ingredients=tranwrd(ingredients,'caramek','caramel');

ingredients=tranwrd(ingredients,'carcmel','caramel');

ingredients=tranwrd(ingredients,'caremel','caramel');

ingredients=tranwrd(ingredients,'carmael','caramel');

ingredients=tranwrd(ingredients,'carmel','caramel');

if (index(ingredients,'caramel col')>0)

or (index(ingredients,'caramel (col')>0)

or (index(ingredients,'color (caramel')>0)

or (index(ingredients,'coloring (caramel')>0)

or (index(ingredients,'caramel food col')>0)

or (index(ingredients,'e150')>0) then caramelcolor=1;

else caramelcolor=0;

* carnauba wax;

ingredients=tranwrd(ingredients,'carnuaba','carnauba wax');

ingredients=tranwrd(ingredients,'canuba','carnauba wax');

ingredients=tranwrd(ingredients,'carruba','carnauba wax');

ingredients=tranwrd(ingredients,'carnuba','carnauba wax');

ingredients=tranwrd(ingredients,'carauba','carnauba wax');

* EXCLUDE THESE;

ingredients=tranwrd(ingredients,'carnaroli','carn_roli');

ingredients=tranwrd(ingredients,'carnation','carn_tion');

if (index(ingredients,'carna')>0)

or (index(ingredients,'auba')>0)

or (index(ingredients,'e903')>0) then carnaubawax=1;

else carnaubawax=0;

run;

data colors;

set blue;

* CREATE INDICATORS FOR APPEARANCE-RELATED SRIAS (0-11),

AND BINARY VARIABLE (1=YES, 0=NO);

appear11 = blue1 + blue2 + green3 + red3 + red40 + yellow5 + yellow6 +

artificialcolor + titaniumdioxide + carnaubawax +

caramelcolor;

if appear11>0 then appear11b=1;

else appear11b=0;

run;

***********

* TEXTURE *

***********;

* EMULSIFIERS;

data lecithin;

set colors;

* lecithin;

ingredients=tranwrd(ingredients,'licithin','lecithin');

ingredients=tranwrd(ingredients,'letcithin','lecithin');

ingredients=tranwrd(ingredients,'lethicin','lecithin');

ingredients=tranwrd(ingredients,'leticin','lecithin');

ingredients=tranwrd(ingredients,'lacithin','lecithin');

ingredients=tranwrd(ingredients,'leicthin','lecithin');

ingredients=tranwrd(ingredients,'leithin','lecithin');

ingredients=tranwrd(ingredients,'soy ecithin','soy lecithin');

if (index(ingredients,'lecithin')>0)

or (index(ingredients,'soy lec')>0)

or (index(ingredients,'soya lec')>0)

or (index(ingredients,'soybean lec')>0)

or (index(ingredients,'sunflower lec')>0)

or (index(ingredients,'e322')>0) then lecithin=1;

else lecithin=0;

* glycerides;

ingredients=tranwrd(ingredients,'monoglcerides','monoglyceride');

ingredients=tranwrd(ingredients,'digelycerides','diglyceride');

ingredients=tranwrd(ingredients,'digiglycerides','diglyceride');

ingredients=tranwrd(ingredients,'digiycerides','diglyceride');

if (index(ingredients,'monogli')>0)

or (index(ingredients,'monogly')>0)

or (index(ingredients,'mono and dig')>0)

or (index(ingredients,'digl')>0)

or (index(ingredients,'e471')>0) then glycerides=1;

else glycerides=0;

* polysorbate;

ingredients=tranwrd(ingredients,'ploysorbate','polysorbate');

ingredients=tranwrd(ingredients,'plysorbate','polysorbate');

ingredients=tranwrd(ingredients,'poilisorbate','polysorbate');

ingredients=tranwrd(ingredients,'polisorbate','polysorbate');

ingredients=tranwrd(ingredients,'poloysorbate','polysorbate');

ingredients=tranwrd(ingredients,'polyorbate','polysorbate');

ingredients=tranwrd(ingredients,'polysobate','polysorbate');

ingredients=tranwrd(ingredients,'ptolysorbate','polysorbate');

if (index(ingredients,'polysor')>0)

or (index(ingredients,'e432')>0)

or (index(ingredients,'e433')>0)

or (index(ingredients,'e434')>0)

or (index(ingredients,'e435')>0)

or (index(ingredients,'e436')>0) then polysorbate=1;

else polysorbate=0;

* sorbitan monostearate;

ingredients=tranwrd(ingredients,'sobitian','sorbitan');

ingredients=tranwrd(ingredients,'sorbatan','sorbitan');

ingredients=tranwrd(ingredients,'sorbibtan','sorbitan');

ingredients=tranwrd(ingredients,'sorbitanmonostearate','sorbitan');

ingredients=tranwrd(ingredients,'sorbitian','sorbitan');

ingredients=tranwrd(ingredients,'sorbiton','sorbitan');

ingredients=tranwrd(ingredients,'soritan','sorbitan');

ingredients=tranwrd(ingredients,'sortitan','sorbitan');

if (index(ingredients,'sorbitan')>0)

or (index(ingredients,'e491')>0) then sorbitanmonostearate=1;

else sorbitanmonostearate=0;

* diphosphate, triphosphate, polyphosphate, pyrophosphate;

ingredients=tranwrd(ingredients,'diph ','diphosphate');

ingredients=tranwrd(ingredients,'triolyphosphate','polyphosphate');

ingredients=tranwrd(ingredients,'triployphosphate','polyphosphate');

ingredients=tranwrd(ingredients,'tripolphosphate','polyphosphate');

ingredients=tranwrd(ingredients,'tripolyohosphate','polyphosphate');

if (index(ingredients,'diphos')>0)

or (index(ingredients,'triphos')>0)

or (index(ingredients,'tripoly')>0)

or (index(ingredients,'polyphos')>0)

or (index(ingredients,'rophos')>0)

or (index(ingredients,'e450')>0)

or (index(ingredients,'e451')>0)

or (index(ingredients,'e452')>0) then phosphates=1;

else phosphates=0;

* polyglycerol polyricinoleate;

ingredients=tranwrd(ingredients,'polygricinoleate','polyricinoleate');

ingredients=tranwrd(ingredients,'polyrricinoleate','polyricinoleate');

ingredients=tranwrd(ingredients,'polyrcinoleate','polyricinoleate');

if (index(ingredients,'polyri')>0)

or (index(ingredients,'e476')>0)

or (index(ingredients,'pgpr')>0)

then polyglycerolpolyricinoleate=1;

else polyglycerolpolyricinoleate=0;

* sodium-stearoyl-2-lactylate;

ingredients=tranwrd(ingredients,'searoyl','stearoyl');

ingredients=tranwrd(ingredients,'slearoyl','stearoyl');

ingredients=tranwrd(ingredients,'sodiumstearoyl','sodium stearoyl');

ingredients=tranwrd(ingredients,'sodiuum','sodium');

ingredients=tranwrd(ingredients,'sodm','sodium');

ingredients=tranwrd(ingredients,'sodum','sodium');

ingredients=tranwrd(ingredients,'staeroyl','stearoyl');

ingredients=tranwrd(ingredients,'staroyl','stearoyl');

ingredients=tranwrd(ingredients,'steaoryl','stearoyl');

ingredients=tranwrd(ingredients,'stearyl','stearoyl');

ingredients=tranwrd(ingredients,'stearyol','stearoyl');

ingredients=tranwrd(ingredients,'stearyollactylate','stearoyl lactylate');

ingredients=tranwrd(ingredients,'steoroyl','stearoyl');

ingredients=tranwrd(ingredients,'steoryl','stearoyl');

ingredients=tranwrd(ingredients,'steqroyl','stearoyl');

ingredients=tranwrd(ingredients,'steroyl','stearoyl');

ingredients=tranwrd(ingredients,'steryol','stearoyl');

ingredients=tranwrd(ingredients,'strearoyl','stearoyl');

ingredients=tranwrd(ingredients,'streroyal','stearoyl');

ingredients=tranwrd(ingredients,'stroyl','stearoyl');

ingredients=tranwrd(ingredients,'styl','stearoyl');

ingredients=tranwrd(ingredients,'styroyl','stearoyl');

ingredients=tranwrd(ingredients,'syeroyl','stearoyl');

if (index(ingredients,'dium stearo')>0)

or (index(ingredients,'e481')>0) then sodiumstearoyl=1;

else sodiumstearoyl=0;

* ammonium phosphatides;

if (index(ingredients,'ammonium phosphatides')>0)

or (index(ingredients,'e442')>0) then ammoniumphosphatides=1;

else ammoniumphosphatides=0;

* sodium and calcium caseinate;

ingredients=tranwrd(ingredients,'sodiumcaseinate','sodium caseinate');

ingredients=tranwrd(ingredients,'soduim','sodium');

if (index(ingredients,'ium cas')>0) then caseinate=1;

else caseinate=0;

run;

data emulsifiers;

set lecithin;

* CREATE INDICATORS FOR EMULSIFIERS (TEXTURE-RELATED SRIAS) (0-9),

AND BINARY VARIABLE (1=YES, 0=NO);

emulsifier9 = lecithin + glycerides + polysorbate +

sorbitanmonostearate + polyglycerolpolyricinoleate +

phosphates + sodiumstearoyl + ammoniumphosphatides +

caseinate;

if emulsifier9>0 then emulsifier=1; else emulsifier=0;

run;

* STABILIZERS, THICKENERS;

data guargum;

set emulsifiers;

* guar gum;

ingredients=tranwrd(ingredients,'guat gum','guar gum');

ingredients=tranwrd(ingredients,'gular gum','guar gum');

ingredients=tranwrd(ingredients,'guiar gum','guar gum');

ingredients=tranwrd(ingredients,'gur gum','guar gum');

ingredients=tranwrd(ingredients,'gura gum','guar gum');

ingredients=tranwrd(ingredients,'gua gum','guar gum');

ingredients=tranwrd(ingredients,'gaur gum','guar gum');

* EXCLUDE THESE;

ingredients=tranwrd(ingredients,'paraguariensis','paragua_iensis');

ingredients=tranwrd(ingredients,'novaguard','novagua_d');

ingredients=tranwrd(ingredients,'jaguar','jagua_');

ingredients=tranwrd(ingredients,'guarantee','gua_antee');

ingredients=tranwrd(ingredients,'guara','gua_a');

ingredients=tranwrd(ingredients,'cytoguard','cytogua_d');

if (index(ingredients,'guar')>0)

or (index(ingredients,'e412')>0) then guargum=1;

else guargum=0;

* carrageenan;

ingredients=tranwrd(ingredients,'carrangeenan','carrageenan');

ingredients=tranwrd(ingredients,'carregeenan','carrageenan');

ingredients=tranwrd(ingredients,'carrgeenan','carrageenan');

ingredients=tranwrd(ingredients,'carrrageenan','carrageenan');

ingredients=tranwrd(ingredients,'caarageenan','carrageenan');

ingredients=tranwrd(ingredients,'carageenan','carrageenan');

ingredients=tranwrd(ingredients,'carageeran','carrageenan');

ingredients=tranwrd(ingredients,'cargeenan','carrageenan');

if (index(ingredients,'carrag')>0)

or (index(ingredients,'e407')>0) then carrag=1;

else carrag=0;

* xanthan gum;

ingredients=tranwrd(ingredients,'xamthan','xanthan');

ingredients=tranwrd(ingredients,'xanathan','xanthan');

ingredients=tranwrd(ingredients,'xanyham','xanthan');

ingredients=tranwrd(ingredients,'xathan','xanthan');

ingredients=tranwrd(ingredients,'xhantan','xanthan');

ingredients=tranwrd(ingredients,'xnathan','xanthan');

ingredients=tranwrd(ingredients,'xnathum','xanthan');

ingredients=tranwrd(ingredients,'xnthan','xanthan');

ingredients=tranwrd(ingredients,'xoathan','xanthan');

* EXCLUDE THESE;

ingredients=tranwrd(ingredients,'astaxanthin','astaxan_hin');

ingredients=tranwrd(ingredients,'canthaxanthin','canthaxan_hin');

ingredients=tranwrd(ingredients,'cantxanthin','canthxan_hin');

if (index(ingredients,'xant')>0)

or (index(ingredients,'e415')>0) then xant=1;

else xant=0;

* locust/carob bean gum;

ingredients=tranwrd(ingredients,'lacust','locust');

ingredients=tranwrd(ingredients,'lcust','locust');

ingredients=tranwrd(ingredients,'lucust','locust');

ingredients=tranwrd(ingredients,'carab','carob');

ingredients=tranwrd(ingredients,'carbo bean','carob bean');

ingredients=tranwrd(ingredients,'carobbean','carob bean');

ingredients=tranwrd(ingredients,'carobean','carob bean');

ingredients=tranwrd(ingredients,'carobg','carob');

ingredients=tranwrd(ingredients,'caroub','carob');

ingredients=tranwrd(ingredients,'carob beam','carob bean');

if (index(ingredients,'locu')>0)

or (index(ingredients,'e410')>0)

or (index(ingredients,'bean gum')>0)

or (index(ingredients,'carob bean gum')>0) then locustcarobbean=1;

else locustcarobbean=0;

* arabic gum and acacia gum;

ingredients=tranwrd(ingredients,'arabac','arabic gum');

ingredients=tranwrd(ingredients,'aca cia','acacia');

ingredients=tranwrd(ingredients,'m acaia','acacia');

ingredients=tranwrd(ingredients,'acaia g','acacia gum');

ingredients=tranwrd(ingredients,'acaicia','acacia');

ingredients=tranwrd(ingredients,'acasia','acacia');

ingredients=tranwrd(ingredients,'achcia','acacia');

ingredients=tranwrd(ingredients,'acoia','acacia');

ingredients=tranwrd(ingredients,'acac','acacia');

ingredients=tranwrd(ingredients,'gumand','gum and');

* EXCLUDE THESE;

ingredients=tranwrd(ingredients,'arabinogalactan','arab_nogalactan');

ingredients=tranwrd(ingredients,'arabinoglactan','arab_noglactan');

ingredients=tranwrd(ingredients,'arabica','arab_ca');

if (index(ingredients,'arabi')>0)

or (index(ingredients,'e414')>0)

or (index(ingredients,'cacia')>0) then acaciagum=1;

else acaciagum=0;

* hydrogenated oils;

ingredients=tranwrd(ingredients,'hydrgntd','hydrogenated');

ingredients=tranwrd(ingredients,'hydrogeanted','hydrogenated');

ingredients=tranwrd(ingredients,'hydrogeated','hydrogenated');

ingredients=tranwrd(ingredients,'hydrogena ted','hydrogenated');

ingredients=tranwrd(ingredients,'hydrogenatd','hydrogenated');

ingredients=tranwrd(ingredients,'hydrogenate','hydrogenated');

ingredients=tranwrd(ingredients,'hydrogenatec','hydrogenated');

ingredients=tranwrd(ingredients,'hydrogenatesoybean','hydrogenated soy');

ingredients=tranwrd(ingredients,'hydrogenerated','hydrogenated');

ingredients=tranwrd(ingredients,'hydrogentaed','hydrogenated');

ingredients=tranwrd(ingredients,'hydrogentated','hydrogenated');

ingredients=tranwrd(ingredients,'hydrogented','hydrogenated');

ingredients=tranwrd(ingredients,'hydrogntd','hydrogenated');

ingredients=tranwrd(ingredients,'hydrogonated','hydrogenated');

ingredients=tranwrd(ingredients,'hydrogrnated','hydrogenated');

ingredients=tranwrd(ingredients,'hydronated','hydrogenated');

ingredients=tranwrd(ingredients,'hydropgenasted','hydrogenated');

* EXCLUDE THESE;

ingredients=tranwrd(ingredients,'non hydrogenated','non hydrog_nated');

ingredients=tranwrd(ingredients,'nonhydrogenated','non hydrog_nated');

if (index(ingredients,'hydrogenated')>0)

or (index(ingredients,'enated v')>0)

or (index(ingredients,'enated s')>0)

or (index(ingredients,'enated p')>0)

or (index(ingredients,'enated c')>0) then hydrogenatedoils=1;

else hydrogenatedoils=0;

* cellulose;

ingredients=tranwrd(ingredients,'celulose','cellulose');

ingredients=tranwrd(ingredients,'celllulose','cellulose');

ingredients=tranwrd(ingredients,'ceullulose','cellulose');

ingredients=tranwrd(ingredients,'ceelulose','cellulose');

ingredients=tranwrd(ingredients,'cullulose','cellulose');

ingredients=tranwrd(ingredients,'methylcyttellulose','methylcellulose');

ingredients=tranwrd(ingredients,'cellelose','cellulose');

ingredients=tranwrd(ingredients,'celleulose','cellulose');

ingredients=tranwrd(ingredients,'celllose','cellulose');

ingredients=tranwrd(ingredients,'cellolose','cellulose');

* EXCLUDE THESE;

ingredients=tranwrd(ingredients,'cellulase','cell_lase');

ingredients=tranwrd(ingredients,'hemicellulose','hemicell_lose');

ingredients=tranwrd(ingredients,'microcellular','microcell_lar');

if (index(ingredients,'cellu')>0)

or (index(ingredients,'e460')>0)

or (index(ingredients,'e461')>0)

or (index(ingredients,'e462')>0)

or (index(ingredients,'e463')>0)

or (index(ingredients,'e464')>0)

or (index(ingredients,'e465')>0)

or (index(ingredients,'e466')>0) then cellulose=1;

else cellulose=0;

run;

data thickeners;

set guargum;

* CREATE INDICATORS FOR THICKENERS (0-7),

AND BINARY VARIABLE (1=YES, 0=NO);

thickener7 = guargum + xant + carrag + locustcarobbean + acaciagum +

hydrogenatedoils + cellulose;

if thickener7>0 then thickener=1; else thickener=0;

run;

* FAT REPLACERS;

data modified;

set thickeners;

* modified starches;

ingredients=tranwrd(ingredients,'unmodified','unmod_fied');

ingredients=tranwrd(ingredients,'commodity','commod_ty');

if (index(ingredients,'modi')>0)

or (index(ingredients,'e1401')>0) then modified=1;

else modified=0;

* alginates;

ingredients=tranwrd(ingredients,'aliginate','alginates');

* EXCLUDE THESE;

ingredients=tranwrd(ingredients,'naturalginger','natural_ginger');

if (index(ingredients,'algin')>0)

or (index(ingredients,'e400')>0)

or (index(ingredients,'e401')>0)

or (index(ingredients,'e402')>0)

or (index(ingredients,'e403')>0)

or (index(ingredients,'e404')>0)

or (index(ingredients,'e405')>0) then alginates=1;

else alginates=0;

* olestra;

if (index(ingredients,'olestra')>0) then olestra=1;

else olestra=0;

* polydextrose;

ingredients=tranwrd(ingredients,'ploydextrose','poly_extrose');

if (index(ingredients,'poly_ex')>0)

or (index(ingredients,'e1200')>0) then polydextrose=1;

else polydextrose=0;

* whey protein concentrate;

ingredients=tranwrd(ingredients,'processedwhey','processed whey');

ingredients=tranwrd(ingredients,'wheyprotein','whey protein');

ingredients=tranwrd(ingredients,'protien','protein');

if (index(ingredients,'whey protein con')>0) then wheyproteincon=1;

else wheyproteincon=0;

run;

data fr;

set modified;

* CREATE INDICATORS FOR FAT REPLACERS (0-5),

AND BINARY VARIABLE (1=YES, 0=NO);

fr5 = modified + alginates + olestra + polydextrose + wheyproteincon;

if fr5>0 then fr=1; else fr=0;

* CREATE INDICATORS FOR ALL TEXTURE-RELATED SRIAS (0-21),

AND BINARY VARIABLE (1=YES, 0=NO);

texture21 = emulsifier9 + thickener7 + fr5;

if texture21>0 then texture=1; else texture=0;

run;

* THE DATA STATEMENT BELOW CREATES INDICATORS REPRESENTING:

1. HOW MANY OF THE FOUR TYPES OF SRIAS ARE IN THE FOOD

(POSSIBLE RANGE 0-4)

2. TOTAL # SRIAS IN THE FOOD (POSSIBLE RANGE 0-64)

3. BINARY VARIABLE REPRESENTING YES/NO TO ANY OF THE 64 SRIAS;

data all;

set fr;

additive4 = sweet + flavor + appear11b + texture;

additive64 = sweet26 + flavor6 + appear11 + texture21;

if additive4>0 then additive=1; else additive=0;

run;

proc freq data=all;

tables highfructose sorbitol mannitol xylitol erythritol maltitol

polyglycitolsyrup canejuice cornsyrupsolids dextrose

glucose maltodextrin maltol maltose ricesyrup fructose dextrin

refinerssyrup fruitjuicecon invertsugar ns20 ns

sucralose saccharin aspart acesu neotame stev nns6 nns

sweet sweet26

artificialflavor naturalflavor disodiumguanylate

autolyzedyeastextract disodiuminosinate

hydrolyzedvegetableprotein flavor flavor6

blue1 blue2 green3 red3 red40 yellow5 yellow6 artificialcolor

titaniumdioxide carnaubawax caramelcolor appear11 appear11b

lecithin glycerides polysorbate sorbitanmonostearate

polyglycerolpolyricinoleate sodiumstearoyl

ammoniumphosphatides caseinate

guargum carrag xant phosphates acaciagum hydrogenatedoils

cellulose locustcarobbean

modified alginates olestra polydextrose wheyproteincon

emulsifier emulsifier9 thickener thickener7 fr fr5

texture texture21

additive additive4 additive64;

run;
